# ﻿A review of the anthidiine bees (Apoidea, Megachilidae) in Thailand

**DOI:** 10.3897/zookeys.1186.95203

**Published:** 2023-12-19

**Authors:** Pakorn Nalinrachatakan, John S. Ascher, Max Kasparek, Prapun Traiyasut, Chawatat Thanoosing, Natapot Warrit

**Affiliations:** 1 Center of Excellence in Biology and Department of Biology, Faculty of Science, Chulalongkorn University, Bangkok 10330, Thailand Chulalongkorn University Bangkok Thailand; 2 Insect Diversity Lab, Department of Biological Sciences, National University of Singapore, 16 Science Drive 4 S3 Level 4, 117558 Singapore, Singapore National University of Singapore Singapore Singapore; 3 Mönchhofstr., 16, 69120 Heidelberg, Germany Unaffiliated Heidelberg Germany; 4 Program in Biology, Faculty of Science, Ubon Ratchathani Rajabhat University, Ubon Ratchathani 34000, Thailand Ubon Ratchathani Rajabhat University Ubon Ratchathani Thailand; 5 Department of Life Sciences, Natural History Museum, Cromwell Road, London SW7 5BD, UK Natural History Museum London United Kingdom

**Keywords:** Pollinator, resin bees, Southeast Asia, taxonomy, wool carder bees

## Abstract

Bees of the tribe Anthidiini (Apoidea: Megachilidae) are notable pollinators consisting of resin bees, wool-carder bees, and cleptoparasitic bees. Twelve anthidiine species were historically reported in Thailand, though the taxonomic information of the group was needed revising. In this study, 165 (97♀, 68♂) anthidiine bee specimens deposited at the Chulalongkorn University Natural History Museum, Thailand, were examined with material obtained from various museum collections. Specimens were principally collected in Thailand with some from Laos and Myanmar. Here, at least eight genera and 15 species of anthidiine bees are recognized: *Anthidiellum* (5), *Bathanthidium* (1), *Eoanthidium* (1), *Euaspis* (4), *Pachyanthidium* (1), *Pseudoanthidium* (1), *Stelis* (1), and *Trachusa* (1). *Dianthidiumchinensis* Wu, 1962, *Eoanthidiumchinensis* (Wu, 1962), *Eoanthidiumsemicarinatum* Pasteels, 1972, and *Eoanthidiumpunjabensis* Gupta & Sharma, 1953 are relegated as junior synonyms of Eoanthidium (Hemidiellum) riparium (Cockerell, 1929), **stat. nov.** Both Anthidiellum (Pycnanthidium) latipes (Bingham, 1897) from Phang Nga and Euaspisaff.wegneri Baker, 1995 from Chumphon were identified as new records for Thailand. Trachusaaff.vietnamensis Flaminio & Quaranta, 2021 from Phitsanulok is a new record for the genus found in Thailand, whereas Pseudoanthidium (Pseudoanthidium) orientale (Bingham, 1897) is a new record for Laos. Annotated comments are provided for some taxa and identification keys for the Thai anthidiine bees is provided.

## ﻿Introduction

Megachilid bees in tribe Anthidiini are robust, usually with yellow maculation and sparse pubescence on the body. The diagnostic characters for the Anthidiini include a short pterostigma (length less than twice of its width), the absence of a median spine on the metanotum, and in many species also by an absence of long hairs on the hind tibial surface (Michener 2007; Gonzalez et al. 2012). Anthidiine bees are cosmopolitan, comprising more than 900 described species worldwide (Ascher and Pickering 2022) that exhibit various nesting strategies within their solitary lifestyles.

The tribe Anthidiini is classified into three groups, based on their nesting material usages: resin users, plant fiber users, and cleptoparasitic species (Fabre 1891; Michener 2007). Because of the variations in morphology, numerous classification systems were hypothesized (see Pasteels 1969; Warncke 1980; Michener 2007; Urban and Moure 2012). Recently, Litman et al. (2016) suggested that many anthidiine genera are paraphyletic based on the results of a molecular phylogeny, the authors classified the anthidiine bees into five monophyletic clades: *Anthidium* group, *Anthodioctes* group, *Dianthidium* group, *Trachusa* group, and *Stelis* group.

Anthidiine bees have been scarcely collected in Thailand, except for the cleptoparasitic *Euaspispolynesia* Vachal, 1903 since its preferred host, Megachile (Callomegachile) disjuncta (Fabricius, 1781), is common. Only 12 species of Anthidiini have been previously recorded in Thailand (Ascher and Pickering 2022), belonging to six genera: *Anthidiellum* (5 species), *Bathanthidium* (1 sp.), *Euaspis* (3 spp.), *Pachyanthidium* (1 sp.), *Pseudoanthidium* (1 sp.), and *Stelis* (1 sp.) (Friese 1925; Cockerell 1929; Dover 1929; Pasteels 1980; Baker 1995; Engel 2009; Tadauchi and Tasen 2009; Niu et al. 2019; Nalinrachatakan et al. 2021b). Nalinrachatakan et al. (2021b) described two new species, one of Anthidiellum (Ranthidiellum) and another of Stelis (Malanthidium), as well as documented their nesting biology.

There are persistent taxonomic difficulties for Thai anthidiines which need revision since many species were only recorded once. For example, the rare endemic resin bee genus AnthidiellumsubgenusRanthidiellum, of which four species were recorded, two were only known from females (see Pagden 1934; Pasteels 1969, 1972; Ascher et al. 2016). *Stelissiamensis* Friese, 1925 was described solely from one male specimen and later synonymized under *Bathanthidiumbinghami* (Friese, 1901) by Niu et al. (2019). Furthermore, a single female of *Dianthidiumriparium* Cockerell, 1929 was synonymized under Anthidiellum (Pycnanthidium) by Soh et al. (2016) since *Dianthidium* is known to be a New World genus. Hence, this study aims to summarize the current status of the anthidiine bees in Thailand by combining museum specimen data with a citizen science database.

## ﻿Material and methods

One hundred and sixty-five anthidiine specimens (97♀, 68♂) were examined in this study. Most of the specimens were collected after 2003 and deposited at the Chulalongkorn University Natural History Museum (**CUNHM**; 71♀, 54♂). Others were on loan from the Department of Entomology and Plant Pathology, Faculty of Agriculture, Chiang Mai University (**CMU**; 1♀), Department of Entomology, Faculty of Agriculture, Kasetsart University (**KKIC**; 3♀), and from the Princess Maha Chakri Sirindhorn Natural History Museum, Faculty of Science, Prince of Songkla University (**PMCS**; 1♀). Specimens in Kumar et al. (2017) and deposited at the Department of Entomology, University of Agricultural Sciences (**UAS**; 10♀, 5♂), Bangalore, India, were also examined. Access to the type specimens and other materials was kindly provided by the Natural History Museum, London (**NHMUK**; 5♀), Naturalis Biodiversity Center: Leiden, Netherlands (**NBC**; 1♀, 2♂), the Royal Belgian Institute of Natural Sciences (**RBINS**; 1♂), Snow Entomological Museum Collection, Lawrence, Kansas, USA (**SEMC**; 5♀, 4♂), Natural History Museum, Berlin, Germany (**ZMB**; 1♂), and the Zoological Survey of India (**ZSI**; 1♂).

Specimens were photographed with two photographic systems. The first system used the Canon 7D Mark II digital camera attached to a Zeiss Stemi 508 stereomicroscope, with a T2-T2 1.6× SLR long-distance microscope lens, controlled via Canon EOS Utility software. The second system used the identical digital camera but was mounted into the Cognisys Stackshot Macro Rail Package system and attached with Canon MP-E 65 mm f/2.8 1-5× macro lens. These sets of photographs were calibrated using AXIOVISION SE64 Rel. 4.9.1 software, for the measurement of the morphological characters of the specimens. All images taken were then post-processed using Adobe Photoshop CC 2018 and Adobe Lightroom CC 2018 software. Other software, including Adobe Illustrator CC 2018, ImageJ, Google Earth Pro, and QGIS (3.16.0) were also used to produce the illustrations, examining small and often overlooked characters, and ascertaining the localities of the samples through mapping.

Male bee specimens were dissected for their genitalia and terminalia examination: i.e., using 3M KOH to clear out muscular artifacts and later preserved in glycerin (adapted from Gonzalez et al. 2012 and Nalinrachatakan et al. 2021b). All terminology used follows Michener and Griswold (1994), Michener et al. (1994), Michener (2007), Engel (2009), and Kasparek (2017). The abbreviations T1, T2, T3, …, S1, S2, S3, … and F1, F2, F3, … are referred to in sequential order of tergum, sternum, and antennal flagellomere, respectively.

In addition to records obtained through specimen examinations, the five Thai Anthidiini taxa were consulted in iNaturalist (2023) to expand spatiotemporal coverage for these bees. All images were identified by one or more of the authors. Due to the inherent uncertainty of identification of species-level taxa likely new to science from images, we do not treat these occurrences in full here, but do comment on the image-based records where pertinent under entries for the set of species could be confirmed by specimens. The records can be accessed through the URL “https://www.inaturalist.org/observations/” followed by its corresponding observation identification number provided in material section.

## ﻿Results

### ﻿Taxonomic account

#### ﻿ Anthidiellum

Taxon classificationAnimaliaHymenopteraMegachilidae

Cockerell, 1904

592819F2-594B-506C-A3A6-458F7B6D9E55

Anthidium (Anthidiellum) Cockerell, 1904: 3. Type species: Trachusastrigata Panzer, 1805, by original designation.

##### Note.

*Anthidiellum* is a small-robust genus that has an arcuate subantennal suture (Fig. 2C), carinated omaulus (see Fig. 2A), open scutoscutellar suture (see Fig. 2B), and presence of a propodeal fovea behind the spiracle. Two distinct subgenera were recognized in Thailand: *Pycnanthidium* Krombein, 1951, with a generally black-yellow integument and a prominent frontal carina on T1; and *Ranthidiellum* Pasteels, 1969, which is slightly larger in comparison, with a general black to reddish brown integument (Fig. 4A, B), and with a general appearance similar to a stingless bee.

#### ﻿ Anthidiellum (Pycnanthidium) smithii

Taxon classificationAnimaliaHymenopteraMegachilidae

(Ritsema, 1874)

1D4392BA-53A5-5F56-BC41-5F91A32B1D10

Fig. 1

Anthidium smithii Ritsema, 1874: 111. (♂) Male holotype from Ambarawan, Java (NBC, not examined).
Anthidium minutissimum Bingham, 1903: 6. (♂) Male holotype from “Biserat, Jalor, Siam” [= Yala province, Thailand] (NHMUK, images examined).
Anthidium javanicum Friese, 1909: 257. (♂) Two syntypes from Buitenzorg [= Bogor, Java], collected by Schmiedeknecht.
Anthidiellum smithii smithii (Ritsema): Pasteels 1972: 89–93, figs 31–39; Soh et al. 2016: 51–54, figs 1A, 2.

##### Material examined.

11 (3♀, 8♂). **Myanmar** (new record): 1♀, Dawei city, Dawei Hospital (13°59.117'N, 98°7.479'E, alt. 4 m), 3 May 2018, N. Warrit et al. (CUNHM: BSRU-AA-6909); **Thailand**: 4♂, Chumpon, Lang Suan District, Ban Suan Phueng, (10°01'N, 99°03'E, alt. 10 m), 15 Jul. 2003, N. Warrit (CUNHM: BSRU-AA-1245–1248); 1♀, Khon Kaen (new record), Phu Wiang District, 26 May 2016, N. Warrit et al. (CUNHM: BSRU-AA-4482); 1♂, Surat Thani, Koh Samui District, 17 Jul 2003, N. Warrit (CUNHM: BSRU-AA-1244); 1♀, 3♂, Ubon Ratchthani (new record), Phu Chong Na Yoi National Park, Trail to National Park Protection unit PorJor5 (14°33'4.35"N, 105°25'36.80"E, alt. 216 m), on *Colonaauriculata* (Desf.) Craib. [Malvaceae], 28 Sep. 2020, N. Warrit et al. (CUNHM: BSRU-AB-1367–1370).

##### Distribution.

Indonesia (Bangka, East Kalimantan, Java, Sumba, Maluku Island), LAOS (Houaphanh), Malaysia (Negiri Sembilan, Penang), Myanmar (Dawei, new record), Philippines (Palawan), Singapore, Thailand (Chaiyaphum, Chiang Mai: Tadauchi and Tasen 2009, Chonburi, Chumpon, Khon Kaen, Nan, Surat Thani, Ubon Ratchathani (new record), Yala).

This species can be rarely found in the Southeast Asian region. A similar species, Anthidiellum (Pycnanthidium) carinatum (Wu, 1962), is known from China (Hainan, Yunnan) and India (Tripura) (see Niu et al. 2016; Sardar et al. 2022).

##### Diagnosis.

Within subgenus Pycnanthidium, this species has a small black body (3.9–5.0 mm) with yellow maculations on all tagmata. It differs from other congeners by its metasomal coloration, i.e., T1 with yellow markings laterally, T2 entirely black, T3–T6 with broad yellow bands, mostly interrupted medially on T3; axilla yellow; broad yellow marginal band on scutellum, medially interrupted; outer surface of the hind tibia and hind basitarsus with longitudinal carinae; black apical comb of S5 in male interrupted medially resembles small notch; gonostylus bifid. However, this species is similar to *A.carinatum* (Wu, 1962) from China, although Niu et al. (2016) suggested subtle differences, primarily related to punctures size and color pattern.

##### Floral association.

*Bidenspilosa* L. (Asteraceae), *Muntingiacalabura* L. (Muntingiaceae) (Soh et al. 2016); *Microcostomentosa* Sm. (Malvaceae) (Pasteels 1972); an individual from Ubon Ratchathani was found collecting pollen on the flower of *Colonaauriculata* (Desf.) Craib. (Malvaceae).

##### Remarks.

*Anthidiellumsmithii* was originally reported in Thailand from Yala province as *A.minutissimum* Bingham, 1903. More than a hundred years later, Tadauchi and Tasen (2009) reported it from Chaiyaphum and Chiang Mai provinces. Variations in color pattern of *A.smithii* are widely recognized, as noted by Pasteels (1972) and Soh et al. (2016).

**Figure 1.**
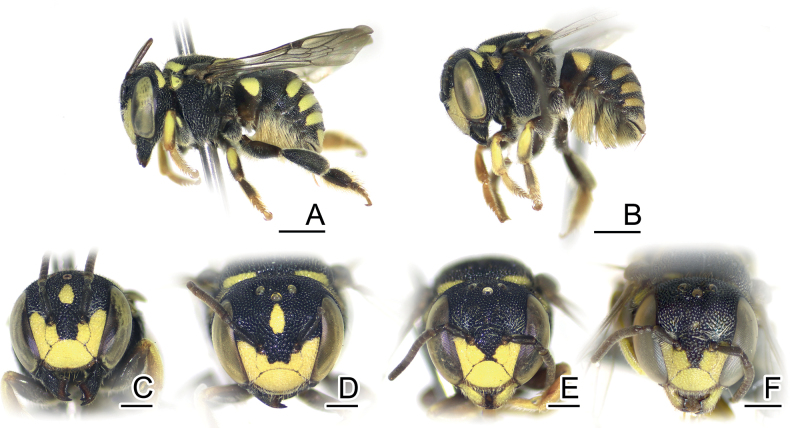
*Anthidiellumsmithii* (Ritsema, 1874) **A**, **B** lateral habitus of females from Myanmar (BSRU-AA-6909) and Khon Kaen (BSRU-AA-4482), Thailand, respectively **C–F** faces of Myanmar female, Khon Kaen female, and Chumporn males (BSRU-AA-1245, 1247). Scale bars: 1 mm (**A, B**); 0.5 mm (**C–F**).

In this study, four males collected at the same time and place in Chumpon Province display varying color patterns. Yellow teardrop markings on the frons are small in two specimens (Fig. 1E, F) (BSRU-AA-1246, BSRU-AA-1247 though the antero-lateral scutal bands are different in size), whereas teardrop marking is expanded in specimens BSRU-AA-1248, and absent in BSRU-AA-1245. All specimens examined have yellow supraclypeal area that is medially interrupted; however, a female from Khon Kaen (BSRU-AA-4482) (Fig. 1D) and one male from Ubon Ratchathani (BSRU-AB-1370) have a yellow marking on its basal area while others do not. Color variation in this species thus can occur in sympatric populations and may be a continuous trait. More discussion on yellow maculation variations is elaborated by Soh et al. (2016).

#### ﻿ Anthidiellum (Pycnanthidium) latipes

Taxon classificationAnimaliaHymenopteraMegachilidae

(Bingham, 1897)

CF60003E-7A19-5107-BF43-23672F37BDEF

Fig. 2

Anthidium latipes Bingham, 1897: 495 (♀). Holotype from “Rangoon” [= Yangon], Myanmar (NHMUK, examined).
Paraanthidium latipes Bingham: Wu 1962: 162–163, fig. 16 (♀, ♂ nov.); Wu et al. 1988: 61 (♀, ♂).
Trachusa (Paraanthidium) latipes (Bingham, 1897): Wu 2006: 180 (♀, ♂).
Anthidiellum (Pycnanthidium) latipes (Bingham, 1897): Niu et al. 2016: 337, 341–343 (♀, ♂).

##### Material examined.

3 (3♀). **Myanmar**: 1♀ holotype, Rangoon [= Yangon, Myanmar], 1–87 Bingham coll., *Anthidiumlatipes* ♀ Bingh Type B.M. TYPE HYM. 17a.1873, Col. C.T. Bingham. 96–30. (NHMUK 014026066); **Thailand**: 2♀, Phang Nga, Kapong District, Tha Na Subdistrict (8°41'38.14"N, 98°24'28.87"E, alt. 29.4 m), 30 Apr. 2020, A. Kaosung (CUNHM, BSRU-AB-0162, 0163).

##### Records from iNaturalist

**(2023).** Myanmar: Yangon, Yangon District, (16°47'12.3"N, 96°08'38.1"E) observed by ‘chimik’ on 25 Apr. 2022. (observation id: 113229246). Thailand: Chiang Mai, Mueang District, Suthep-Pui (18°49'00.5"N, 98°55'26.8"E, accuracy 240 m) observed by ‘jackychiangmai’ on 9 Apr. 2023 (observation id: 154207614), and on 13 Apr. 2023 (observation id: 154700134 and 154702585).

##### Distribution.

China (Yunnan), Myanmar (Yangon), Thailand (Chiang Mai (new record from iNaturalist 2023), Phang Nga (new record)).

##### Diagnosis.

*Anthidiellumlatipes* can be assigned to a group of Asian *Pycnanthidium* which includes *A.butarsis* Griswold, 2001, *A.ramakrishnae* (Cockerell, 1919), *A.rasorium* (Smith, 1875), *A.coronum* (Wu, 2004), and *A.cornu* Tran & Engel, 2023. The group contains medium-sized bees without carina on their hindlegs (Griswold 2001; Niu et al. 2016; see also Tran et al. 2023).

##### Floral association.

**Figure 2.**
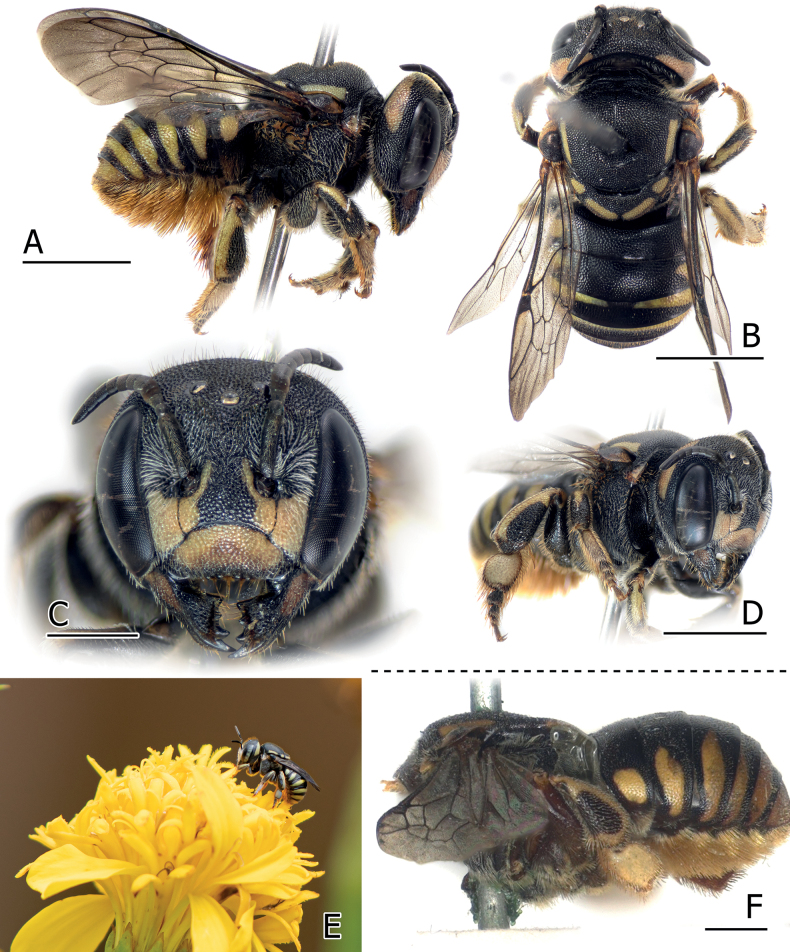
Female of *Anthidiellumlatipes* (Bingham, 1897) from Phang Nga, Thailand (BSRU-AB-0162) (**A–E**), and the female holotype of *A.latipes* from Yangon, Myanmar (NHMUK 014026066: picture modified from NHMUK data portal) (**F**) **A** lateral habitus **B** dorsal habitus **C** face **D** lateral angle showing the omaulus and hind legs **E** female wandering on the marigold flower (*Tageteserecta* L.), photographed by Andaman Kaosung **F** lateral habitus. Scale bars: 2 mm (**A, B, D**); 1 mm (**C, F**).

Marigold (*Tageteserecta* L., Asteraceae, see Fig. 2E) and also yardlong bean (*Vignaunguiculate* (L.), Fabaceae).

##### Remarks.

The knowledge on the Asian *Pycnanthidium* is relatively scant due to the limited material and the damages in type specimens such as in *Anthidiellumramakrishnae* (Griswold, 2001), and with the materials of *A.coronum* (discussed below). The female holotype of *A.latipes* from Myanmar is also not in good condition, the head and most of the legs were missing while the mesosoma and metasoma were glued together.

There is a possibility that *A.coronum* (Wu, 2004) is a junior synonym of *A.latipes*, as the color patterns on the supraclypeal area (Fig. 2C), hindlegs (Fig. 2D), and the shape of enlarged hind basitarsus, with ~ 1.3 length/width ratio, are more or less similar. In addition, the Thai specimens and a specimen recorded from Myanmar (through iNaturalist) share similar maculation on the supraclypeal area as seen in *A.coronum*, including the presence of mesad process running along the upper rim of the antennal socket.

#### ﻿ Anthidiellum (Ranthidiellum) apicepilosum

Taxon classificationAnimaliaHymenopteraMegachilidae

(Dover, 1929)

01BF2C31-11F0-54B5-BA98-D944697DD364

Fig. 3

Dianthidium apicepilosum Dover, 1929: 55 (♀). Holotype from “Khao Ram, Siam” [= Nakhon Si Thammarat, Thailand] (NHMUK, examined); photograph available at https://data.nhm.ac.uk/object/17eba820-1a7d-4175-a9f8-f367b04dbf94/1632960000000).
Dianthidium apicepilosum Dover: Pagden 1934: 490–492 (♂ nov.).
Anthidiellum (Ranthidiellum) apicepilosum (Dover): Pasteels 1969: 48–49.
Anthidiellum (Rhanthidiellum [sic!]) apicepilosum (Dover): Pasteels 1972 (redescription): 103–106, unjustified emendation of Ranthidiellum Pasteels, 1969.

##### Material examined.

1♀. Khao Ram, Siam [= **Thailand**: Nakhon Si Thammarat: Ronpibun, Khao Ramrome], 750–1200 [possibly altitude], 24 Feb. 1922, *Anthidiumapicepilosum* Dover, 1926 (holotype NHMUK: 014026685).

##### Distribution.

Malaysia (Negeri Sembilan, Penang, Selangor), Thailand (Nakhon Si Thammarat). The species is rarely found, hence the records are based on the original designation (Dover 1929) and the additional report of their nest (Pagden 1934)

##### Diagnosis.

*Anthidiellumapicepilosum* has a black body with distinct brownish coloration disrupted. Since only the female is known, the most comparable characters include the following: clypeus, scape, lower paraocular area, tegula, axilla, and the margin of scutellum brownish; scutum black; wing base conspicuously dark brown, clearly contrasting to apical hyaline parts on 1^st^ submarginal cells; T1–T5 with reddish to brownish ferruginous apical band that becomes wider on the rear segments; T6 black; the rear of the metasoma covered with yellowish hairs; foreleg light brownish, generally brighter than in midleg and hindleg, which are almost black on their tibia and basitarsus. According to Pagden (1934) and Pasteels (1972), the male is superficially similar to the female but has lighter ferruginous leg parts especially on midleg and hindleg; S5 with a marginal black comb (> 60 teeth), gonoforceps bifid as in other *Ranthidiellum* species.

##### Remarks.

This is the first species of *Ranthidiellum* that has been documented for its nesting biology (Pagden 1934). In morphology, the species is very close to *Anthidiellumrufomaculatum* (Cameron, 1902) with a minor difference in that the female holotype of “*Protoanthidiumrufomaculatum*” [= *Anthidiellumrufomaculatum*, female from Kuching, Sarawak, Malaysia] has more minute paraocular marks, more slender marginal bands on its axilla and scutellum, and a reddish black translucent band present basally on the black integument of T1–T5. However, to confirm the status, there is no certain evidence that the males described by Pagden (1934) are the exact *Anthidiellumapicepilosum*, even though the locality of Bakit Kutu, Selangor, is adjacent to the Negeri Sembilan, where the female paratype was caught (further discussion in Nalinrachatakan et al. 2021b).

**Figure 3.**
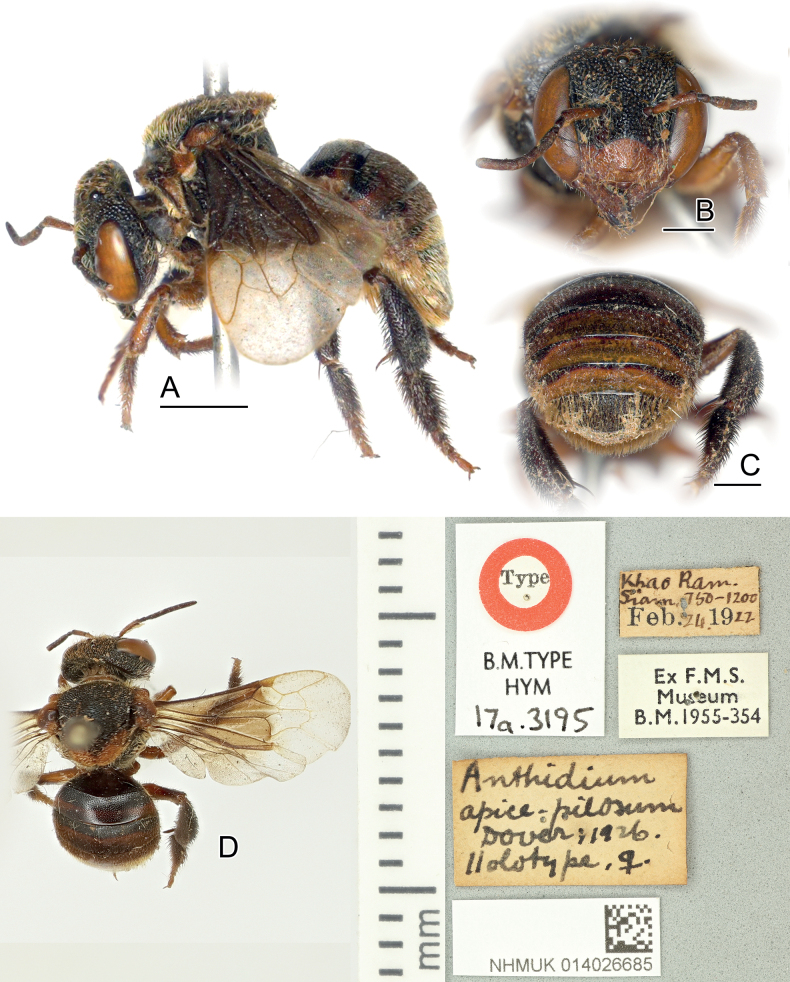
Female holotype of *Anthidiellumapicepilosum* (Dover, 1929) (NHMUK: 014026685) **A** lateral habitus **B** face **C** rear view of metasoma showing T6 **D** dorsal view and labels, modified from NHMUK data portal. Scale bars: 2 mm (**A**); 1 mm (**B, C**).

#### ﻿ Anthidiellum (Ranthidiellum) ignotum

Taxon classificationAnimaliaHymenopteraMegachilidae

Engel, 2009

0BD827F4-FE0D-5AF3-BA72-D95AA35B0A5A

Fig. 4A

Anthidiellum ignotum Engel, 2009: 30–34, figs 1–3. (♀) Holotype from Sakaerat Environmental Research Area, Nakhon Ratchasima Province, Thailand (SEMC, not examined).
Anthidiellum ignotum Engel: Soh et al. 2016: 55 (♀); Nalinrachatakan et al. 2021b: 164–167, figs 2, 4 (right), 5 (right) (♀, ♂ nov.).

##### Material examined.

(6♀, 1♂). Same specimens as in Nalinrachatakan et al. (2021b).

##### Record from iNaturalist

**(2023).** Thailand: Chiang Mai, Mueang District, Suthep Subdistrict (18°49'0.47"N, 98°55'26.81"E) uploaded by ‘jackychiangmai’ on 27 Oct. 2022 (observation id: 140223648).

##### Distribution.

Thailand (Chiang Mai, Nakhon Ratchasima, Phayao). The species is rare and appears to be endemic.

##### Floral association.

Plant family Amaranthaceae (possibly *Achyranthesaspera* L., commonly known as devil’s horsewhip), shown in the iNaturalist observation noted above. Also, the bee must mobilize plant resin as do other *Ranthidiellum* species (Pagden 1932; Pasteels 1972; Nalinrachatakan et al. 2021b).

##### Remarks.

*Anthidiellumignotum* has distinct sexual dimorphism in which the male particularly had its base coloration brighter, with a noticeable black facial mark. More details on the presence of *Ranthidiellum* species in Thailand and its variation were discussed in Nalinrachatakan et al. (2021b).

**Figure 4.**
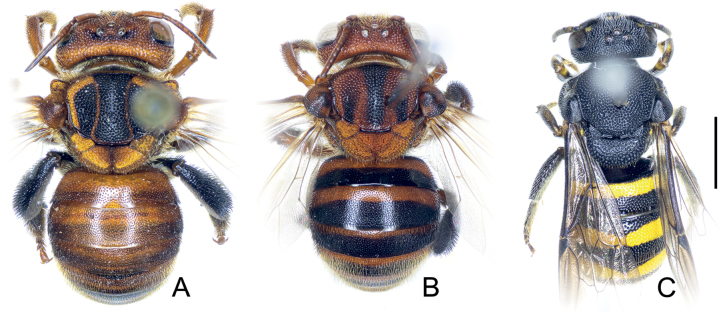
Comparing specimens of Anthidiellum (Ranthidiellum) recently collected from Thailand, and its cleptoparasite **A** female *Anthidiellumignotum* Engel, 2009 (BSRU-AA-1249) **B** female paratype of *Anthidiellumphuchongense* Nalinrachatakan & Warrit, 2021 (BSRU-AB-0159) **C** male paratype of cleptoparasite *Stelisflavofuscinular* Nalinrachatakan & Warrit, 2021 (BSRU-AB-0156). Scale bar: 2 mm.

#### ﻿ Anthidiellum (Ranthidiellum) phuchongense

Taxon classificationAnimaliaHymenopteraMegachilidae

Nalinrachatakan & Warrit, 2021

87DA9166-5AC1-591B-83F8-68F97ADE4798

Fig. 4B

Anthidiellum (Ranthidiellum) phuchongensis Nalinrachatakan & Warrit in Nalinrachatakan et al. 2021b: 167–171, see figs 3, 4 (left), 5 (left). (♀, ♂) Male holotype and female paratypes from Ubon Ratchathani, Thailand.

##### Material examined.

(5♀, 1♂). Same specimens as in Nalinrachatakan et al. (2021b). Holotype was transferred to NHMUK in April 2023..

##### Distribution.

Thailand (Ubon Ratchathani). From a survey in other adjacent national parks in Ubon Ratchathani, there is evidence that *Ranthidiellum* is present through an abandoned nest with collapsed structures (i.e., resin became opaque, whitish, and the entrance apically fractured).

##### Floral association.

Mobilizing the resin of plants in the family Dipterocarpaceae, possibly *Dipterocarpusobtusifolius* Teijsm. ex Miq., which is broadly distributed along their nesting habitats.

##### Bee cleptoparasites.

*Stelisflavofuscinular* Nalinrachatakan & Warrit, 2021.

##### Remarks.

The species-group name *phuchongensis* is changed to *phuchongense* following a mandatory change for gender agreement under ICZN article 34.2. The species was discovered to build its nest in a dipterocarp forest in Phu Chong Na Yoy National Park, in a preexisting hole near water stream. Their nesting structures are unique, with a distinct downwardly curved, resinous, translucent tube. Further details of its nesting biology and morphology variations were discussed in Nalinrachatakan et al. (2021b).

#### ﻿ Bathanthidium

Taxon classificationAnimaliaHymenopteraMegachilidae

Mavromovstakis, 1953

212F85FB-FADA-503B-98E0-F6547EEDD557

Bathanthidium Mavromoustakis, 1953: 837. Type species: Dianthidiumbifoveolatum Alfken, 1937, by original designation.

##### Note.

**Figure 5.**
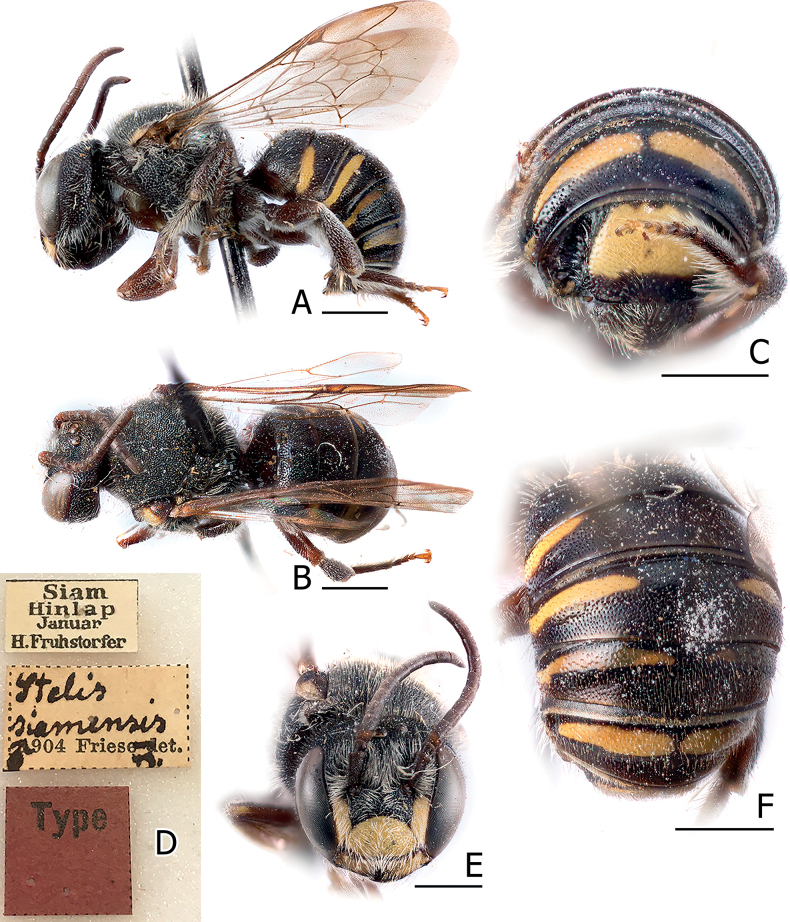
Type specimen of *Stelissiamensis* Friese, 1904, male, from Nan province, Thailand, which was recently synonymized with Bathanthidium (Manthidium) binghami (Friese, 1901) **A** lateral view **B** dorsal view **C** posterior angle of metasoma **D** original label **E** face **F** dorsal of metasoma. Scale bars: 1 mm.

*Bathanthidium* is an Asiatic genus consisting of small to medium-sized species that are mostly found in China. They come with almost black body and distinct yellow maculation (see Fig. 5); face without juxta-antennal carina (Fig. 5E), preoccipital ridge and omaulus not carinate; if omaular carina present, then it does not continue to the venter; presence of propodeal fovea behind propodeal spiracle. The genus was revised by Niu et al. (2019).

#### ﻿ Bathanthidium (Manthidium) binghami

Taxon classificationAnimaliaHymenopteraMegachilidae

(Friese, 1901)

131ED9B8-87B5-5432-BEA5-3291FCA0DFCC

Fig. 5

Anthidium fraternum Bingham, 1897 (nec Pérez 1895): 495 (♀). Holotype from Tenasserim, Myanmar, image also examined in NHMUK under https://data.nhm.ac.uk/media/02e0fc5c-8359-4414-89a1-7d0a53aeded3.
Anthidium binghami Friese, 1901: 224, replacement name for Anthidiumfraternum Bingham, 1897.
Manthidium binghami (Friese, 1901): Pasteels 1969: 43.
Stelis siamensis Friese, 1925: 40 (♂). Holotype from “Siam bei Hinlap” [= Nan province, Thailand] (ZMB, examined).
Paraanthidium concavum Wu, 1962: 164 (♂). Holotype from China, Yunnan, Xishuangbanna (IZCAS: Institute of Zoology, Chinese Academy of Sciences, images examined).
Trachusa (Paraanthidium) concavum (Wu, 1962): Wu 2006: 174, ♂ (key), 184, ♂ (redescription), fig. 100a–e.
Bathanthidium (Manthidium) binghami (Friese, 1901): Rasmussen and Ascher 2008: 30; Niu et al. 2019: 106, fig. 8A–H; Sardar et al. 2022: 78, fig. 6.

##### Material examined.

(2♂). **India**: 1♂, West Bengal, Buxa Tiger Reserve, 22 miles, East Damanpur (26°37.067'N, 89°33.633'E), 27 Mar. 2019, A. Rameshkumar (ZSI) as in Sadar et al. (2022). **Thailand**: 1♂, Siam [= Thailand], Hinlap [= Nan province, “Hinlap” must refer to the area of “Baan Hinlap”, or “Huai Hinlap reservoir” as currently named (not in Chaiyaphum province) in Pua district, Sila lang Subdistrict], Januar [= January], H. Fruhstorfer, *Stelissiamensis*, ♂, 1904, Friese det., Type (ZMB).

##### Records from iNaturalist

**(2023).** Thailand: Chiang Mai Province, Chiang Dao District, (19°24'44.3"N, 98°55'17.3"E) observed by ‘charlieglasser’ on 23 Mar 2023 (observation id: 160344574 and 160340826).

##### Distribution.

China (Yunnan), India (Sikkim, West Bengal), Myanmar (Tenasserim), Thailand (Chiang Mai (new record from iNaturalist 2023), Nan), Laos (Luang Prabang).

##### Diagnosis.

*Bathanthidiumbinghami* has a robust, small to medium-sized body with black integument disrupted by striking yellow markings. The species is distinctly separated from its congeners by the combination of the follows: yellow on its mandible, clypeus, and paraocular area that do not exceed beyond the antennal socket plane; narrow yellow stripe laterally on T2–T5, while tending to abut together on the rear segment; yellow stripe on T6 and also T7 in male; rounded omaulus; T6 (also in the smaller male T7) sub-truncate, with distinct median elevation that extends its apical margin (Fig. 5C, F; Niu et al. 2019: fig. 8E).

##### Remarks.

*Bathanthidium* was revised by Niu et al. (2019), where *Stelissiamensis* Friese, 1925, historically collected from Nan, Thailand, was synonymized under *B.binghami*. Niu et al. (2019) also thoroughly provided pictures of the female holotype of “*Paraanthidiumconcavum*” in comparison to other species. Sadar et al. (2022) pointed out the problematic documentation of its distribution (erroneously recorded for India) while confirming that *B.binghami* was found in India. The species displays the unique sub-truncate apical terga in both sexes.

Through personal communication with Mr. Charles H. Glasser, who provided the iNaturalist records, we know that the bee inhabits farmland cultivated by the indigenous people of Lisu tribe.

#### ﻿ Eoanthidium

Taxon classificationAnimaliaHymenopteraMegachilidae

Popov, 1950

94E02BD3-7139-56A7-AEEB-79F6E2E58C4E

Dianthidium (Eoanthidium) Popov, 1950: 316. Type species: Anthidiuminsulare Morawitz, 1873, by original designation.
Eoanthidium (Eoanthidiellum) Pasteels, 1969: 51. Type species: Anthidiumelongatum Friese, 1897 = Anthidiumclypeare Morawitz, 1873, by original designation.

##### Note.

**Figure 6.**
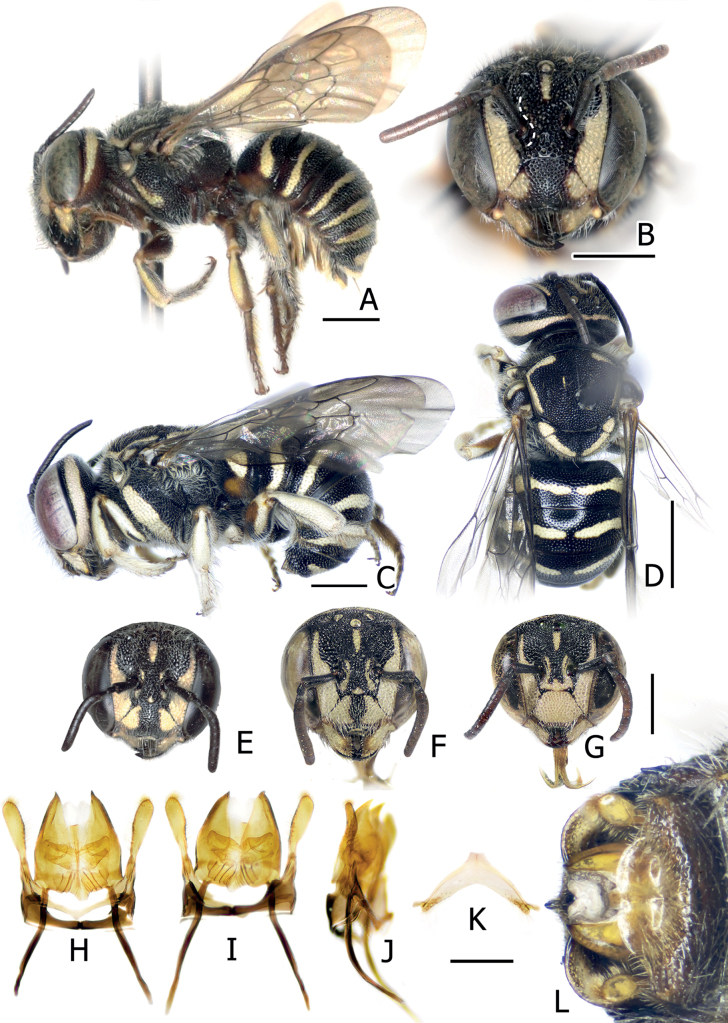
Eoanthidium (Hemidiellum) riparium (Cockerell, 1929). Female holotype of *Dianthidiumriparium* Cockerell, 1929 (syn.) from Thailand (**A, B**). Male from Thailand (BSRU-AB-4360) (**C, D**). Female from Laos (BMNH-ENT-2017-196 (ACQ)) (**E**), Female from Laos (BSRU-AA-1224) (**F**), male from Laos (BSRU-AA-1236) (**G–K**), and male from Thailand (BSRU-AB-4358) (**L**) **H–J** male genitalia in dorsal, ventral, and lateral view **K** male S8 **L** apical sterna of male in ventral view. Scale bars: 2 mm (**D**); 1 mm (**A–C, E–G**); 0.5 mm (**H–L**).

An old-world genus which is mostly discernable from other genera by its more slender, striking black-yellow body, with a distinct juxta-antennal carina (Fig. 6B), rounded preoccipital ridge, carinated omaulus (see Fig. 6A, C), and rounded scutellum (Fig. 6B). Male sterna lack apical comb, penis valve enlarged, and pointed apically. The genus comprises four subgenera with 21 species in the Afrotropical, Palaearctic and Indo-Malayan regions (Kasparek and Griswold 2021). This work focuses on the subgenus Hemidiellum which has only one species to date. The bee subgenus Hemidiellum has a relatively small body length (~ 6–7 mm; Fig. 6) compared to its congeners, with the combination of characters as follow: omaular carina complete; distinct juxta-antennal carina; subantennal suture almost straight; upper margin of keirotrichiate area of hind tibia curved, not carinated; T4–T6 laterally with small tubercles in both sexes; arolia present. In addition to the type species, Gupta (1993) previously assigned *Eo.punjabense* Gupta & Sharma, 1993 to the subgenus Hemidiellum (see also Kasparek and Griswold 2021).

#### ﻿ Eoanthidium (Hemidiellum) riparium

Taxon classificationAnimaliaHymenopteraMegachilidae

(Cockerell, 1929) comb. nov.

7ED8CE2D-4236-539A-95D9-B81628011588

Figs 6 , 7 , 8

Dianthidium riparium Cockerell, 1929: 204 (♀). Holotype from Nan, Thailand (NHMUK, examined).
Dianthidium chinensis Wu, 1962: 167–168, figs 22–26 (♂) (syn. nov.). Type from Yunnan, Xishuangbanna, 9 Apr 1955.
Eoanthidium (Hemidiellum) semicarinatum Pasteels, 1972: 112–116 (♀, ♂) (syn. nov.). Female holotype and male paratypes from Pondicherry State, Karikal, India (NBC, examined).
Eoanthidium (Hemidiellum) punjabensis Gupta & Sharma in Gupta 1993: 37–39, fig. figs 65–77, 79 (♂) (syn. nov.). Male holotype from Pathankot, Punjab, 4 Jun 1991.
Eoanthidium (Hemidiellum) punjabense Gupta & Sharma in Gupta 1993: 37–39, figs 65–77, 79, mandatory change for gender agreement.
Eoanthidium (Eoanthidiums. str.)chinensis (Wu, 1962): Wu, 2006: 134, fig. 66 (syn. nov.).

##### Material examined.

39 (16♀, 23♂). **India**: Karnataka: Bangalore, GKVK, 1♀, 2 Apr. 1982, Ghorepade, 1♂, 15 Apr. 2013, Girish, (UAS); Mysore, 1♀, 19 Apr. 2009, 2♀, 16 Apr. 2009, 1♂, 5 Apr. 2009, Dhanyavathi. (UAS); 1♂, Mandya, 1 May 2014, Veereshkumar (UAS; same specimens as Kumar et al. 2017); 1♂, Coimbatore, 3 Mar. 1950, P. Susai Nathan [*Eoanthidiumsemicarinatum* Past. D.B. Baker det. 1982 / D.&M. Baker collection KUNHM#2004-en-004 / SEMC0975139] (SEMC25); 1♂, Madras State Coimbatore (alt. 1,400 m) Apr. 1962, P. Susai Nathan [*E.semicarinatum* det. Pasteels 1969 / PARATYPE / R.M.N.H.B. 24.136] (RBINS113); 1♂, Hisar, 15 May 1986, A. Rahman [D.&M. Baker collection KUNHM#2004-en-004 / SEMC0975140] (SEMC28); 1♀, Karnataka Malaksamudra Tank, 2 Mar. 1984, K. Ghopada [B21 / Ghorpade collection Bangalore / E. (Hemidiellum) semicarinatum det. C.G. Michener / SEMC1321747 KUNHM-ENT] (SEMC41); Pondicherry State Karikal, Mar. 1962, P. Susai Nathan, 1♀ [*E.semicarinatum* n. sp. Pasteels det. 1969 / HOLOTYPE / RMNH.INS 943212) (NBC001), 1♂ [*E.semicarinatum* n. sp. J.Pasteels det. 1969 / ALLOTYPE / PARATYPE / RMNHS.INS 943188] (NBC033), 1♂ [*E.semicarinatum* n. sp. J.Pasteels det. 1969 / PARATYPE / RMNHS.INS 943189] (NBC034); **Laos** (new record): Champasak, Si Phan Don, 3♀, 11♂, Don Det, 20 Jan. 2015, N. Warrit et al. (CUNHM: BSRU-AA-1220–1222, 1224–1226, 1228–1230, 1232–1234, 1236–1237); 1♀, KHONG ISLAND [= Don Khong], 25 Oct. 2008, D.W. Baldock, E. Popov *Hemidiellum* Pasteels *riparium* (Cckll.) det. Risch, 2008 (NHMUK: BMNH-ENT-2017-196 (ACQ)); **Myanmar** (new record): 1♀, Dawei city (13°50.933'N, 98°9.647'E, alt. 15 m), 3 May. 2018, N. Warrit et al. (CUNHM: BSRU-AA-6896); **Pakistan**: Punjab, Lahore, 2 May 1979, P.H.B. Baker, 1♂, [*E.semicarinatum* Past. D.B. Baker det. 1982 / D.&M. Baker collection KUNHM#2004-en-004 / SEMC0975141] (SEMC26), 1♂, [D.&M. Baker collection KUNHM#2004-en-004 / SEMC0975140] (SEMC27), 4♀, [D.&M. Baker collection KUNHM#2004-en-004 / SEMC0975143–0975146] (SEMC29–32); **Thailand**: 1♀, type, nan. Siam Jan. 7. (Cockerell) [= Nan province, 7 Jan, year is not indicated on the label, but Cockerell’s work was published in 1929], *Dianthidiumriparium* TYPE: Ckll., B.M. TYPe HYM. 17?? [?? = may be “01” but is difficult to read] 1939, Brit. Mus. 1933-567 (NHMUK 014026126); 1♂, Chiang Mai, Chiang Dao District, Chiang Dao Wildlife Sanctuary (19°24'53.2506"N, 98°54'53.2218"E, alt. 541 m) specimen from TIGER project T-5776, 19/25 Feb 2008, Songkran & Apichart (CUNHM: BSRU-AB-4358); 1♂, Lampang, Mueang Pan District, Chae Son National Park (18°49'44.2488"N, 99°28'15.1026"E, alt. 509 m), specimen from TIGER project T-5413, 7/14 Apr 2008, Boonruen & Acharaporn (CUNHM: BSRU-AB-4360).

##### Distribution.

China (Yunnan, new record), India (Haryana: Hisar, Karnataka: Bangalore, Koppala, Mysore, Mandya, Tamil Nadu: Karikal, Coimbatore, Punjab: Pathankot), Laos (Champasak, new record), Myanmar (Dawei, new record), Pakistan (Punjab: Lahore), Thailand (Chiang Mai (new record), Lampang (new record), Nan (new record)).

##### Diagnosis.

The species exhibits pale yellow maculation, remarkably on supraclypeal area (which is reduced medially into a unique shape or absent (Fig. 6), as shown for Laotian, Myanmarese, Thai holotype of *Dianthidiumriparium* Cockerell, 1929 and Chinese *D.chinensis* Wu, 1962), two paramedian yellow stripes on the scutum, and wide yellow bands on all terga which is often a little disrupted at the median on T1–T5 and also T6 in males. In males, T7 with a broadly rounded lateral lobe and a small median notch with cutting end. Genitalia as in Fig. 6H–J, gonostylus in ventral with inner swollen base, apodeme of penis valves extremely extended.

##### Floral associations.

The record of Chinese element (Wu 1962) mentioned “Eupatoreae sp.” (today recognized as Eupatorieae, Asteraceae) from China. Noteworthy, the group includes the globally invasive “Tropical whiteweed” (*Ageratumconyzoides* L.), that also widely distributed in South China and locally used as a biocontrol plant to enhance the productivity of farmland (Huang et al. 2011). The study from India by Gorain et al. (2012) reported the visitation of *Eoanthidiumpunjabense* on "ghaf" tree (*Prosopiscineraria* (L.) Druce (Fabaceae)).

##### Remarks.

Although the specimens from China, Laos, Myanmar, and Thailand are different in their coloration compared to the type bearing the name Eoanthidium (Hemidiellum) semicarinatum, some characters and male genitalia are unique among the genus, and obviously comparable (see also the figures in Wu 1962; Pasteels 1972; Kumar et al. 2017). The female of “*Dianthidiumriparium*” was described by Cockerell (1929), but he did not refer to its juxta-antennal carina, the main character of the genus *Eoanthidium*, and *Eoanthidium* was designated later (Popov 1950). Likewise, the description and the illustrations of Eoanthidium (Eoanthidium) chinensis (Wu, 1962) (Wu 1962: figs 23–26; 2006: fig. 66) and photographs of further material held in IZCAS provided by Ze-Qing Niu are adequate to synonymize this taxon with *Eo.riparium*.

**Figure 7.**
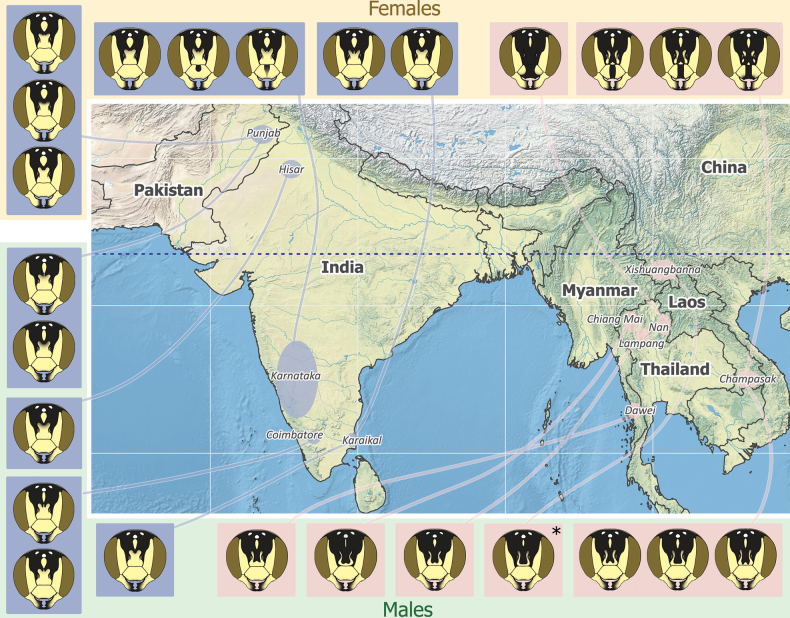
Illustration of Eoanthidium (Hemidiellum) riparium (Cockerell, 1929) facial maculation mapped according to their geographic locations (blue boxes indicate Indian-Pakistani morphs, reddish boxes indicate Indochina morphs; a morph marked with asterisk is illustrated based on Wu 1962: fig. 22).

When compared with “*Eo.semicarinatum*” specimens from India and Pakistan, it is evident that the individuals from Southeast Asia and China (Yunnan) are larger and darker, and tend to come with a reduction in pale yellow facial maculation in supraclypeal area, frons, and on mesepisternum, scutum, and scutellum (Fig. 7). Most of the paramedian band on the scutum is obscure, narrow, and not connected to the anterolateral mark, while the yellow mark on female hindlegs is not fully extended as in Indian and Pakistani specimens, thus, making the apical part of tibia, basitarsus, and the most of tarsi black. The pattern in most of the Laotian and Myanmarese specimens have a distinct reduction of the supraclypeal mark in the middle. A specimen from Khong Island (Fig. 6E) shows more reduction, evidenced by the disruption in the middle of the stripe below the antennal socket, coupled with an additional apical disruption noticed on the clypeus. For the female holotype of *Dianthidiumriparium* from Nan, Thailand, the maculation is more reduced, clearly absent in its supraclypeal area (Fig. 6B), and thus has a strong disruption on the clypeus.

**Figure 8.**
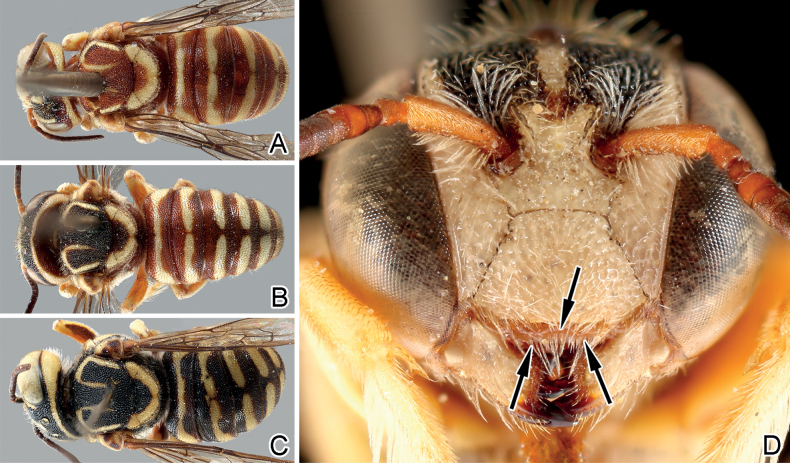
Male of *Eoanthidiumriparium* (Cockerell, 1929) from different regions **A–C** dorsal view of individuals from South India (Karnakata), Pakistan (Panjab), and North India (Hisar, Haryana) respectively **D** face of a male *Eoanthidiumriparium* from Pakistan (SEMC27). Note the change in the ground color from black (**A**) across reddish brown on terga and black on scutum (**B**) to entirely reddish brown (**C**). Also, note the shape of the reddish apical margin of the clypeus (black arrows) which is similar to the drawing of Gupta (1993: fig. 65) for *Eo.punjabense*.

Individuals from the eastern part of the distribution (China, Laos, Myanmar, and Thailand) have a black background color of the integument with yellow markings (Fig. 8). The same is true for the populations in southern India. However, a male from northern India (Hisar) has a reddish brown background color of meso- and metasoma; the ground color of the head is black (face) and reddish brown (vertex). Individuals from Pakistan take an intermediate position, characterized by a scutum with a black background and the abdominal terga with a reddish brown background color (Fig. 8). No sexual differences were noted in the distribution of this pattern, i.e., both sexes are paler in Pakistan (4 females, 2 males) and north India (1 male), while the eastern populations are dark in both sexes.

Additionally, specimens from India and Pakistan have a much larger paramedian mark on the scutum, often extending to connect with the anterolateral mark, and generally they display more extensive maculations. The female almost has a fully yellow hindleg, sometimes with the black left on the tarsi and parts of basitarsus. Such individuals with richer yellow maculation are typical for Pakistan. Some females from southern India (including those shown by Kumar et al. 2017: figs 7, 8) have a greater reduction in the yellow clypeal mark, resulting in a complete black stripe in the median area, whereas other specimens come with fully yellow without any black disruption.

For some West Palaearctic *Eoanthidium* and *Rhodanthidium* species, Kasparek (2019b, 2020, 2021) reported regional variations in the color pattern and discussed the possibility that the “darker” forms might be a result of adaptation to solar radiation. While in some cases, color variation follows geographical clines, there seems to be reproductive isolation between pale and dark forms in other cases. We observed a clear geographical pattern of color variants in *Eo.riparium*, but also intermediate forms in Pakistan (see Fig. 8), indicating that there is no reproductive isolation.

In addition, *Eoanthidiumpunjabense* Gupta & Sharma, 1993 is established here as a new synonym of *Eo.riparium*. Gupta (1993) noted that these two species are distinguished by the form of the apical margin of the clypeus, the shape of genitalia, color pattern of the integument and body size. While Gupta (1993) solely relied on Pasteels’ (1972) description, the larger material examined by us as well as the examination of Pasteels’ type material enabled a better understanding of the range of variation. The reddish apical margin of the clypeus is crenulated and somewhat irregularly formed. Our material includes one male with two protrusions (Fig. 8D), very similar to those shown by Gupta (1993: fig. 65). With respect to differences in genital morphology, Gupta (1993) may have been misguided by the incomplete drawings by Pasteels (1972). Body size of *Eo.punjabense* was found to be within the variability range of *Eo.riparium*.

#### ﻿ Euaspis

Taxon classificationAnimaliaHymenopteraMegachilidae

Gerstaecker, 1858

94133010-032A-520F-A75F-72AA4D7673EC

Euaspis Gerstaecker, 1858: 460. Type species: Thynnusabdominalis Fabricius, 1793, by original designation.
Dilobopeltis Fairmaire, 1858: 266. Type species: Dilobopeltisfuscipennis Fairmaire, 1858 = Thynnusabdominalis Fabricius, 1793, by original designation.
Parevaspis Ritsema, 1874: 71. Type species: Parevaspisbasalis Ritsema, 1874, by designation of Sandhouse 1943: 585.

##### Note.

**Figure 9.**
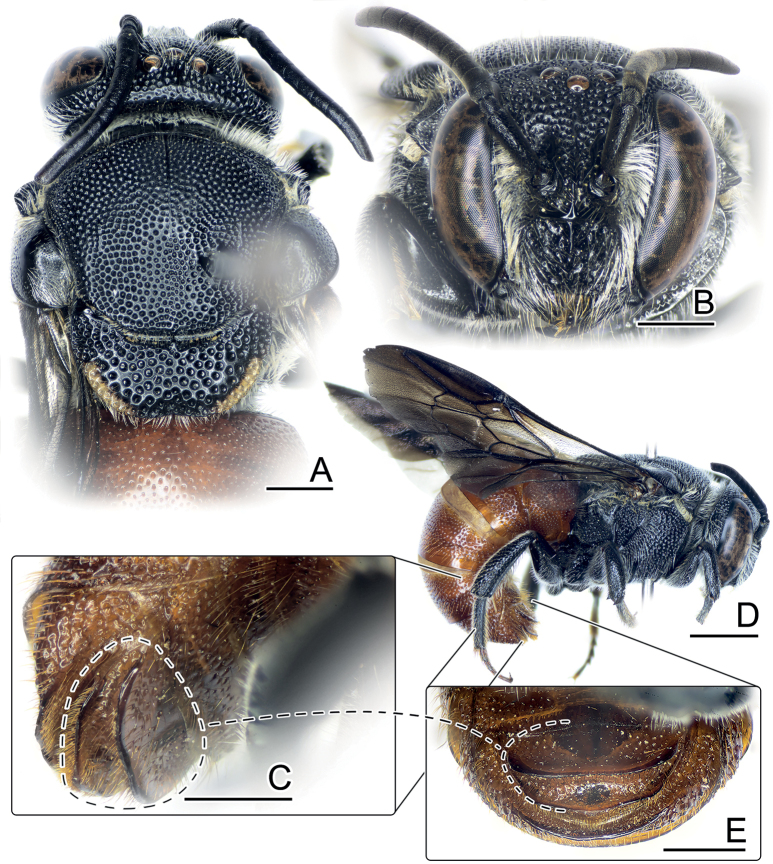
Females of *Euaspisaequicarinata* Pasteels, 1980 (BSRU-AB-4120) **A** mesosoma including the scutellum **B** face **C, E** S6 **D** lateral habitus. Scale bars: 2 mm (**D**); 1 mm (**A–C, E**).

As a cleptoparasitic bee, *Euaspis* has a distinct median longitudinal carina (Fig. 9B), juxta-antennal carina, and often comes with reddish metasoma. Baker (1995) revised the Asiatic species and Tran et al. (2016) noted and discussed three species from Vietnam. Additional material is still crucially required to prove and justify some problematic species in this genus.

#### ﻿ Euaspis aequicarinata

Taxon classificationAnimaliaHymenopteraMegachilidae

Pasteels, 1980

3BA49C93-9A33-562A-841A-CF802D8BC397

Fig. 9

Euaspis aequicarinata Pasteels, 1980: 78 (♀, ♂). Female holotype from Kalabankan, Sabah, Malaysia (image in NHMUK examined under https://data.nhm.ac.uk/media/80bdf262-c729-42ef-bf3a-3180eb81ceb2).
Euaspis aequicarinata Pasteels: Baker 1995: 289, 290, fig. 13; Soh et al. 2016: 57; Tran et al. 2016: 516, fig. 13.

##### Material examined.

(1♀, 2♂). **Thailand**: 1♀, Chiang Mai (new record), Chom Thong District, Ban Luang Subdistrict, Doi Inthanon National Park, Ban Mae Klang Luang. (18°32'17.9"N, 98°32'49.6"E, alt. 1,057 m), 30 Aug. 2021, on *Coleusscutellarioides* (L.) Benth. [Lamiaceae], T. Srimaneeyanon et al. (CUNHM: BSRU-AB-4120); 2♂, Phayao (new record), Mueang District, Maeka Subdistrict, Phayao University (19°1'31.45"N, 99°53'24.17"E, alt. 558 m), 1 Jun 2012, W. Suwannarak et al. (CUNHM: BSRU-AA-4445, 4462).

##### Distribution.

China (Yunnan), Indonesia (Java), Laos (Vientiane), Malaysia (Negeri Sembilan, Borneo: Sabah, Sarawak), Thailand (Chiang Mai (new record), Nakhon Ratchasima, Phayao (new record), Surat Thani)), Vietnam (Kon Tum, Hoa Binh).

##### Diagnosis.

**Figure 10.**
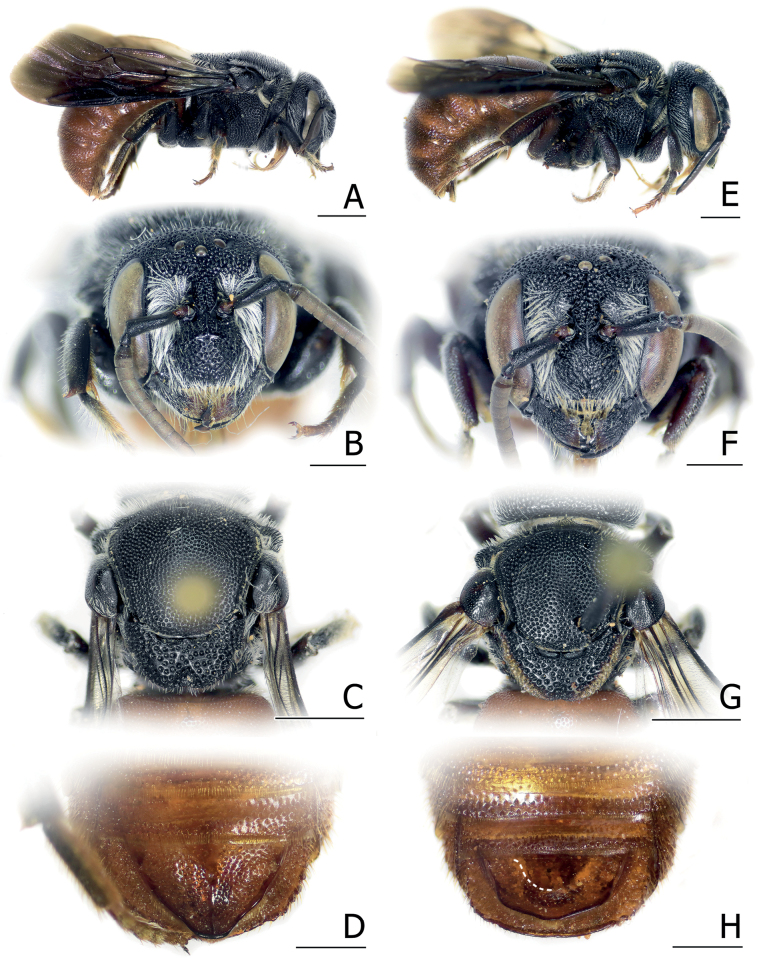
Females of *Euaspispolynesia* Vachal, 1904 (BSRU-AA-4453) (**A**–**D**) and *Euaspisstrandi* Meyer, 1922 (BSRU-AA-4470) (**E–H**) **A, E** lateral habitus **B, F** face **C, G** mesosoma including the scutellum **D, H** S6, with a white dash line indicating boundary of the median elevated area in the left. Scale bars: 2 mm (**A, C, E, G**); 1 mm (**B, D, F, H**).

Typically for *Euaspis*, *Eu.aequicarinata* has a black body with a reddish metasoma, and a median carina and a juxta-antennal carina are present on its face. This is the only species that has a distinct longitudinal carina on the clypeus, while the sculptures are confluent. Pale yellow patches are found on the lateral margin of scutellum and posterior margin of axilla (absent on axilla for female in this study, in contrast to the monochrome pictures in Baker (1995: fig. 30); compared to *Eu.strandi*, the band is more restrict and more yellowish. Female S6 obtuse, with distinct elevated basal platform (Fig. 10C, E). Male S5 with median subcircular hyaline area and median tooth, genitalia with an apical lamina which is longer than 2× its width.

##### Floral associations.

*Coleusscutellarioides* (L.) Benth. (Lamiaceae).

##### Remarks.

As mentioned in Soh et al. (2016), the status of male *Eu.aequicarinata* is not confirmed since the male allotype was designated from Surat Thani, Thailand, while originally, the female holotype is from Sabah (in Borneo), Malaysia. For this reason, there is currently no proof that the male is associated with the holotype regardless of the fact that they are similar in clypeal form and mesosomal maculation. Also, Pasteels (1980) did not mention any male genitalia or associated structures. Baker (1995) subsequently rectified the erroneous type locality in his key for Oriental *Euaspis* species, noting that *Eu.aequicarinata* should have an apical lamina longer than twice its width.

#### ﻿ Euaspis polynesia

Taxon classificationAnimaliaHymenopteraMegachilidae

Vachal, 1904

87D6D3F2-FD52-57AB-8162-2B2051D83C6D

Fig. 10A–D

Stelis abdominalis Smith, 1858 (nec Fabricius 1793): 7. (♂) Holotype from Celebes [= Sulawesi] (OUMNH: Oxford University Museum, not examined).
Euaspis polyesia Vachal, 1903a: 97. (♀ nov., ♂), incorrectly labeled (Baker 1995), replacement name of Stelisabdominalis Smith, 1858.
Euaspis polynesia Vachal, 1903b: 173, justified emendation.
Euaspis smithii Friese, 1904: 137, unnecessary replacement name.
Parevapis impressus Vierick, 1924: 745. (♀, ♂) Male holotype and female allotype from Surigao, Mindanao (USNM: United States National Museum, not examined).
Euaspis basalis chinensis Cockerell, 1930: 50. (♀, ♂). Female type and male cotype from Foochow, China (NHMUK, not examined).
Euaspis (Parevapis) polynesia Vachal: Popov 1933: 377.
Euaspis (Parevapis) polyesia Vachal: Pasteels 1980: 76–89, incorrectly labeled.
Euaspis polynesia Vachal: Baker 1995: 286–289; Soh et al. 2016: 55–56, figs 1, 6; Tran et al. 2016: 517–518, figs 7–10; Ghosh et al. 2023: 193–196, fig. 1.

##### Material examined.

43 (20♀, 23♂). **Thailand**: 1♀, Chainat (new record) [with obscured label] (KKIC); 1♀, Chanthaburi, Makam District, 25 May 2015, N. Chattanabun (CUNHM: BSRU-AA-4458); Chiang Mai, Chom Thong District, Ban Luang Subdistrict, Doi Inthanon National Park, Ban Mae Klang Luang, Tourist Station, 1♀, (18°32'2.8"N, 98°32'55"E, alt. 1015 m), 16 Jun. 2019, N. Warrit et al., on *Cupheahyssopifolia* K. [Lythraceae] (CUNHM: BSRU-AA-7927); 1♀, (18°32'28.4"N, 98°32'57.2"E, alt. 1020 m), 26 Oct. 2020, T. Srimaneeyanon et al. (CUNHM: BSRU-AB-1372), Chiang Mai, Chom Thong District, Ban Luang Subdistrict, Doi Inthanon National Park, Ban Mae Klang Luang. 2♂, (18°32'12.17"N, 98°32'48.99"E, alt. 1,056 m), 17 Feb. 2021, Srimaneeyanon et al. (CUNHM: BSRU-AB-2810, 2843), 1♀ 2♂, (18°32'29.7"N, 98°32'1.2"E, alt. 1,033 m), 01:00–05:00PM, 7 May 2021, T. Srimaneeyanon et al. (CUNHM: ♀ BSRU-AB-3459, ♂ BSRU-AB-3548, 3550), 1♀ 2♂, (18°32'29.6"N, 98°33'01.4"E, alt. 1,012 m), 30 Aug. 2021, T. Srimaneeyanon et al. (CUNHM: ♀ BSRU-AB-4153, ♂ BSRU-AB-4154, 4155). Chiang Mai, Chom Thong District, Ban Luang Subdistrict, Doi Inthanon National Park, Mae Klang Waterfall, 1♀, (18°29'40.70"N, 98°40'01.95"E, alt. 330 m), 18 Feb. 2021, T. Srimaneeyanon et al. (CUNHM: BSRU-AB-3056), 1♀, (18°29'33.2"N, 98°40'13.1"E, alt. 319 m), 8 May 2021, T. Srimaneeyanon et al. (CUNHM: BSRU-AB-3320). 1♀, Chiang Mai, Mae Rim District, Pong Yaeng Subdistrict, Queen Sirikit Botanic Garden, 21 Aug. 2016, Aerial net, N. Chatthanabun (CUNHM: BSRU-AB-1782); Kanchanaburi (new record), 1♀, Tha Sao Dist., Hellfire pass interpretive centre (14°21'4.1472"N, 98°57'23.5476"E, alt. 240 m), 17 Dec. 2021, S. Deowanish et al. (CUNHM: BSRU-AB-5493). 1♀, 1♂, Sai Yok District, Wang Krachae Subdistrict (14°11'6.5724"N, 99°3'6.9258"E, alt. 102.3 m), 24 Jun. 2016, N. Warrit et al. (CUNHM: BSRU-AA-4480, 4483); 1♂, Mukdahan (new record), Mueang District (16°34'11.4630"N, 104°43'47.1426"E, alt. 139 m), 18 Jan. 2017, N. Warrit et al. (CUNHM: BSRU-AA-4903); Nakhon Pathom (new record), 1♀, Kamphaeng Saen District, 8 Jul. 2003, Subat (KKIC). 1♀, 21 Nov 2002, Pornwat (KKIC). 2♂, (13°44'58.3908"N, 99°52'33.1242"E, alt. 14 m), 10 Jul. 2015, N. Warrit et al. (CUNHM: BSRU-AA4466, 4467). 2♀, KU Kamphaengsaen Campus, Insect Park (14°02'18.1500"N, 99°58'56.5016"E, alt. 3 m), 29 Jul. 2015, N. Warrit et al. (CUNHM: BSRU-AA-4476, 4477); 6♂, Phayao (new record), Mueang District, Maeka Subdistrict, Phayao University (19°1'31.45"N, 99°53'24.17"E, alt. 558 m), 1 Jun. 2012, W. Suwannarak et al. (CUNHM: BSRU-AA-4446, 4448, 4450, 4463, 4464, 4465); 1♀, Phetchabun (new record), Lomsak District, Bungkla Subdistrict (18°15'N, 103°58'E, alt. 162 m), 18 Oct. 2009, K. Attasopa & P. Phukphume (CUNHM: BSRU-AA-4442); 1♀, Phetchaburi (new record), Kang Kra Chan District, 18 Apr. 2012, C. Rungsri (CUNHM: BSRU-AA-4443); Ratchaburi (new record), 1♀, Jom Bung District, 26 May 2012, N. Warrit & W. Suwannaruk (CUNHM: BSRU-AA-4453). 2♂, Ratchaburi, Suan Phueng District, Pasutara resort (13°31'5.9226"N, 99°20'51.6366"E, alt. 104.71 m), 2 Aug. 2019, P. Senawong et al. (CUNHM: BSRU-AB-0764, 0765); Saraburi (new record), 1♂, Kaeng Khoi District, Chula-Saraburi (14°31'3"N, 101°1'41"E, alt. 43 m), 15 Aug. 2015, N. Warrit et al. (CUNHM: BSRU-AA-4481). 1♀, (14°31'23.4300"N, 101°1'43.5216"E, alt. 52.89 m), 13 Oct. 2018, N. Warrit et al. (CUNHM: BSRU-AB-0154); 2♂, Trang, Na Yong District (7°33'8.0892"N, 99°46'33.6072"E, alt. 24 m), 11 Jun. 2015, N. Warrit et al. (CUNHM: BSRU-AA-4457, 4459); Ubonratchathani (new record), 1♂, Khueng Nai District, Ko Ae Subdistrict, Ubon Rachathani Rajabhat Univ. Faculty of Agriculture, 30 Aug. 2020, P. Traiyasut et. al. (CUNHM: BSRU-AB-1704), 1♂, Na Chaluai District, Phu Chong Na Yoi National Park (14°26'4.98"N, 105°15'31.04"E, alt. 269 m), 23 Jan. 2015, N. Warrit et. al. (CUNHM: BSRU-AA-4460), 1♀, Na Chaluai District, Phu Chong Na Yoi National Park, Pa Lan Pa Chad (14°26'5.36"N, 105°15'39.92"E, alt. 280 m), 27–29 Sep. 2020, P. Traiyasut et. al. (CUNHM: BSRU-AB-1704).

##### Distribution.

China (Anhui, Fujian, Gansu, Guangdong, Hebei, Hunan, Jiangsu, Jiangxi, Shangdong, Xizang, Yunnan, Zhejiang), Hong Kong, Indonesia (Bali, Bangka Island, Engano Island, Java, Maluku Islands [Ambon, Buru, Kai islands], Sebesi Island, Sumatra, Sulawesi), India (Arunachal Pradesh), Japan (Okinawa Prefecture), Laos (Xiengkhouang), Malaysia (Kedah, Kelantan, Melaka, Penang, Perak, Selangor), Myanmar (Shan State, Tenasserim, Yangon), Nepal (Kathmandu), Philippines (Luzon, Mindanao), Singapore, Taiwan (Pingtung), Thailand (Chiang Mai, Chainat (new record), Kanchanaburi (new record), Loei, Mukdahan (new record), Nakhon Pathom (new record), Pattani, Phayao (new record), Phetchabun (new record), Phetchaburi (new record), Ratchaburi (new record), Saraburi (new record), Satun, Songkhla (new record), Surat Thani, Trang (new record), Ubon Ratchathani (new record)), Vietnam (Bak Kan, Dak Lak, Dak Nong, Dien Bien, Hoa Binh, Phu Tho, Son La, Thanh Hoa, Vinh Phuc).

Most of the previous records were documented by Baker (1995) and Pasteels (1980). The species is widely distributed in Eastern Asia, especially in South-east Asia, where Soh et al. (2016), Tran et al. (2016), and Ghosh et al. (2023) reported additional distribution records.

##### Diagnosis.

This *Euaspis* species has an entirely reddish metasoma, while the prosoma and mesosoma are all black; face with longitudinal carina and a median longitudinal ridge; clypeus with uniform punctation; punctures on the scutellum looser and coarser than on the scutum; scutellum large, strongly produced posteriorly, apicomedially with a depressed area; female S6 acute, with a median carina, without a distinct basal area (Fig. 10D); male S6 without emargination at the margin; male genitalia with the apical lamina with a length less than twice its width.

##### Floral associations.

A female collected from Chiang Mai was wandering on the inflorescences of “Tropical whiteweed” *Ageratumconyzoides* L. (Asteraceae), “Black-Jack” *Bidenspilosa* (L.) Benth. (Asteraceae), and “Mexican heather” *Cupheahyssopifolia* K. (Lythraceae). For Singapore, Soh et al. (2016) reported that *Eu.polynesia* visits the flowers of *Averrhoacarambola* L. (Oxalidaceae), *Grammatophyllumspeciosum* Blume (Orchidaceae), *Muntingiacalabura* L. (Muntingiaceae), and *Premnaserratifolia* L. (Lamiaceae). They also mention *Cordiacylindristachya* (Ruiz & Pav.) Roem. & Schult. (Boraginaceae) and *Antigononleptopus* Hook. & Arn. (Polygonaceae), of which the latter genus was also given by Baker (1995). The recent study from India by Ghosh et al. (2023) found *Eu.polynesia* nectaring on *Fagopyrumesculentum* Moench (Polygonaceae).

##### Host-parasite relationship.

Bingham (1897) noted a single male of *Eu.polynesia* accessing the nest of *Megachiledisjuncta* (Fabricius, 1781). Since the female generally takes on the parasitizing task, this must not be the direct act of the invasion for parasitisation, but the reason remains unknown.

##### Remarks.

*Euaspispolynesia* is the most common anthidiine bees in Thailand, exhibiting a size range, with the females ranging 9.0–13.1 mm and the males 6.2–12.1 mm. As a cleptoparasitic bee, its occurrence seems to follow the distribution of its hosts, especially *Megachiledisjuncta* (see Baker 1995), which is very common and widely distributed in Thailand. Thus *M.disjuncta* is a megachilid species with most individuals curated at the CUNHM (i.e., 102 specimens curated, from 905 Megachilidae specimens). All 11 *Euaspis* localities in the CUNHM database (Nalinrachatakan et al. 2021a) have been associated with bees from the genus *Megachile* (at least 7 subgenera were identified). *Aethomegachile* (8 localities) and *Callomegachile* (8) contributed the most, e.g., M. (Ca.) umbripennis (6), M. (Ca.) disjuncta (5), and M. (A.) laticeps (6). Other notable species belonged to M. (Creightonella) fraterna (5), and another cleptoparasitic bee genus *Coelioxys* was also found in eight of 11 occasions. This information shows that both *Euaspis* and *Coelioxys* may have a wide range of their candidate host, and there is the possibility of overlap or of having an evolutionary pressure on each other that cannot be ignored, as both genera were reported to come with different brood-parasitizing strategies (Litman et al. 2016; Litman 2019).

A probable new species of *Euaspis* from Singapore (Soh et al. 2016) is superficially similar to *Eu.polynesia*, but mostly differs in the terminalia, apical terga coloration, and with a pale mark on the scutellum margin, while their genitalia are superficially similar. Further studies and molecular evidence are required to resolve this taxonomic conundrum.

#### ﻿ Euaspis strandi

Taxon classificationAnimaliaHymenopteraMegachilidae

Meyer, 1922

51D564EC-BE3D-5D72-8DD7-6F13458DE6AE

Fig. 10E–H

Euaspis (Parevaspis) strandi Meyer, 1922: 236, 239 (♀, ♂, syntypes, male selected as lectotype by Baker 1995). Type locality erroneously noted as “Sikkim”, and Baker (1995) corrected it to be Mindanao, Philippines (ZMB, not examined).
Parevaspis bakeri Vierick, 1924: 745 (♂). Holotype from Kolambugan, Mindanao, Philippines (USNM: United States National Museum, not examined).
Euaspis strandi (Meyer): Baker 1995: 291, 293.

##### Material examined.

(2♀). **Thailand**: Phayao (new record), Mueang District, Maeka Subdistrict, Phayao University (19°1'31.45"N, 99°53'24.17"E, alt. 558 m), 1 Jun. 2012, W. Suwannarak et al. (CUNHM: BSRU-AA-4444, 4470).

##### Distribution.

China (Yunnan, “Kinpin”: Wu 1962: 168 as *Parevaspisbakeri*), Thailand (Nakhon Ratchasima, Phayao: new record), Philippines (Mindanao).

##### Diagnosis.

*Euaspisstrandi* has a reddish metasoma, whereas the rest of the body is black, with a remarkable pale yellow stripe on the mesonotum (i.e., axilla and scutellum with pale yellow marginal band); clypeus with coarse, somewhat irregular punctures (Fig. 10F); punctures on scutellum looser and coarser than on scutum; scutellum large, produced posteriorly with a small shallow median emargination; female apical margin of S6 obtuse, with an enlarged basal platform which contributes ~ 1/2 of the sternal length (Fig. 10H); male was purposed by Viereck (1924) as without mesosomal yellow stripe, apical lamina of gonoforceps with a length of more than twice its width.

##### Floral associations.

*Sindorasiamensis* Teijsm. ex Miq. (Fabaceae) is associated with the female collected from Nakhon Ratchasima, Thailand (Baker 1995).

##### Remarks.

In Thailand, *Eu.strandi* was reported from Sakaerat, Nakhon Ratchasima province in 1995 (Baker 1995). Two females examined from Phayao province were quite large (11.4 mm and 11.5 mm) compared with *Eu.polynesia*, which varies considerably in size.

The female individual was not observed in this study. Previously, two male specimens had been designated, the first one by Meyer (1922) as syntype together with a female specimen, and the second by Viereck (1924) as *Parevaspisstrandi*. Both specimens were redescribed and discussed by Baker (1995), and the locality of Meyer’s syntype was corrected and the male was selected to be a lectotype. Therefore, the validity of the female identity is still ambiguous, also mentioned in Baker (1995): syntypes were mislabeled, collected without any notes to confirm that they come from the same locality, and are doubtfully paired since the notable character does not match, i.e., an absence of the marginal mark on scutellum and axilla, which is noticeable in the female.

#### ﻿ Euaspis aff. wegneri

Taxon classificationAnimaliaHymenopteraMegachilidae

Baker, 1995

98D04A9A-7BFA-53EA-A3C4-44EA8AFF2AA2

Fig. 11

Euaspis wegneri Baker, 1995: 290, figs 24, 31 (♀). Holotype from “BATJAN” [= Bacan, north Maluku, Indonesia] (NBC, not examined).

##### Material examined.

1♀. **Thailand**: Chumporn (new record?), Sawi District, Na Sak Subdistrict (10°10'10.7"N, 98°56'50.5"E), 1 Jun. 2021, Suntaree Kanchananiyom. (PMCS: SK-BSRU-0068 [association number with CUNHM]).

##### Distribution.

Indonesia (Bacan province in north Maluku [= Batjan (in Dutch) in Baker (1995)], Thailand (Chumporn, new record?).

##### Diagnosis.

**Figure 11.**
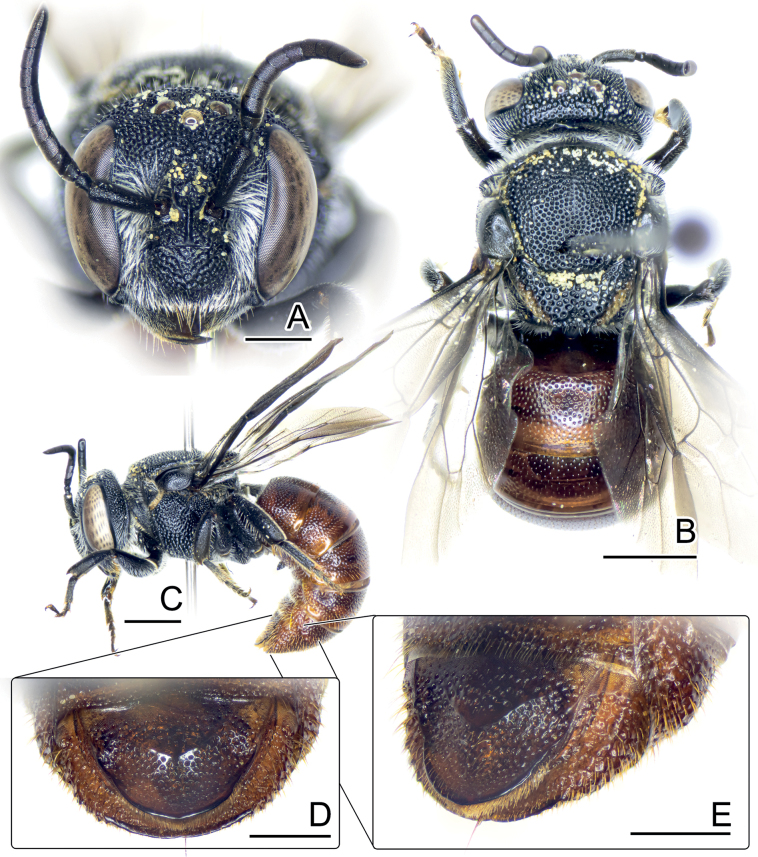
Female of Euaspisaff.wegneri Baker, 1995 **A** face **B** dorsal habitus **C** lateral habitus **D, E** S6. Scale bars: 2 mm (**B, C**); 1 mm (**D, E**); 0.5 mm (**A**).

This female Euaspisaff.wegneri has a typical black body and reddish metasoma, with a pale yellow stripe on the mesonotum (Fig. 11B) resembling *Eu.strandi* (pale yellow marginal band on scutellum, more minute on axilla). Clypeus moderate in size, somewhat confused punctures, while the punctures on the scutellum are coarser than on scutum. Female S6 (Fig. 11D, E) subacute with obscured basal platform contributing almost half of the length of S6 while the apical half of S6 forming faint median carina, lateral margin with small, obscured, blunt teeth. In contrast, yellow maculation in mesosoma is absent in the holotype of *Eu.wegneri* (see Baker 1995: figs 24, 28, 31) and has more fine and dense punctures on the scutum and scutellum, while S6 seems to be more acute.

##### Floral associations.

Unknown.

##### Remarks.

*Euaspiswegneri* has been described on the basis of a single female and has never been reported after that. The holotype of *Eu.wegneri* represented with monochrome digitization in Baker (1995: figs 24, 28, 31, for S6, face, and mesonotum, respectively) arguably has a black mesosoma, with more fine and dense punctures on scutellum compared to scutum. Here, we decided to put this Thai specimen as Eu.aff.wegneri since the other characters are comparable especially on S6 which is distinctive for each *Euaspis* species. Other characters can be considered variations, although S6 of the holotype seems to be more acute. With the possibly new species noted in Soh et al. (2016) (also noted in the *Eu.polynesia* section above), we consider that examination of more specimens coupled with molecular analyses are needed in order to resolve the true identity of these enigmatic specimens.

#### ﻿ Pachyanthidium

Taxon classificationAnimaliaHymenopteraMegachilidae

Friese, 1905

71E42AD6-8B75-5673-A376-3B8B7B1AF200

Anthidium (Pachyanthidium) Friese, 1905: 66–75. Type species: Anthidiumbicolor Lepeletier, 1841 designated by Cockerell 1920: 298.
Pachyanthidium Friese: Cockerell 1930: 45.

##### Note.

**Figure 12.**
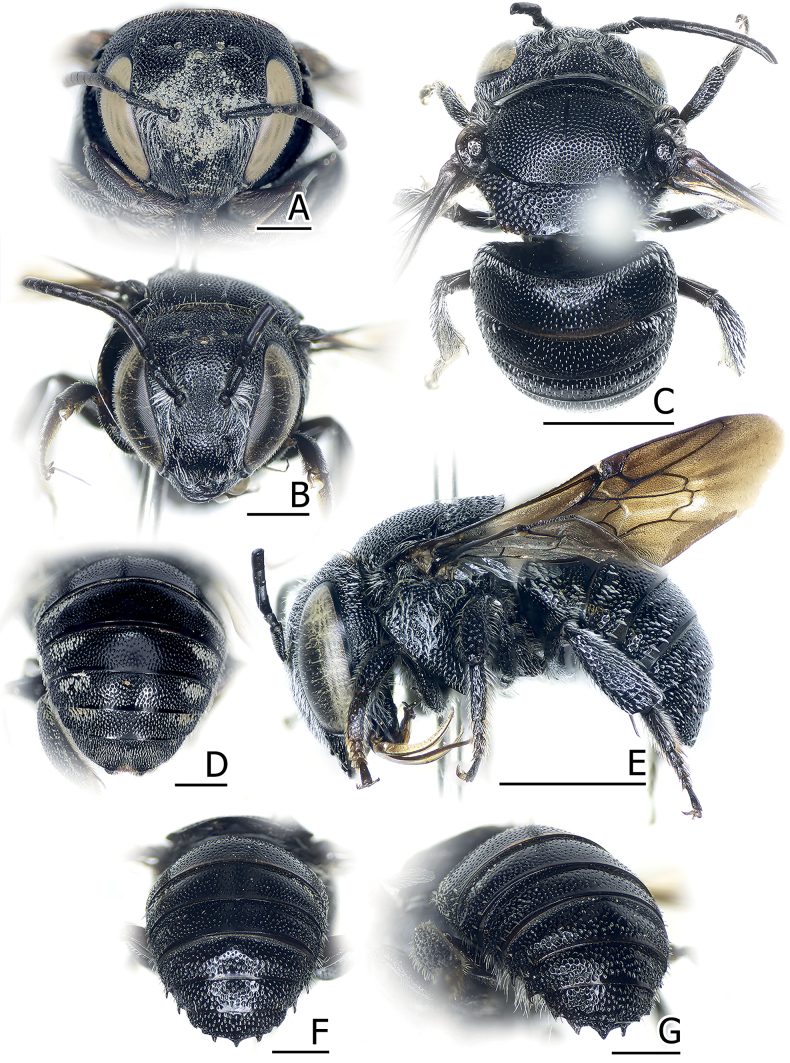
Pachyanthidium (Trichanthidium) lachrymosum (Smith, 1879) **A** female face **B** male face **C** male dorsal habitus **D** female metasoma **E** male lateral habitus **F, G** male metasoma. Scale bars: 2 mm (**C, E**); 1 mm (**A, B, D, F, G**).

This genus can be easily distinguished by its explicit robust body, closed scutoscutellar suture (Fig. 12C), and lamellated preoccipital carina, omaular carina, and scutellum (see Fig. 12C, E). Eardley and Griswold (2017) revised 16 Afrotropical species of this genus from 18 described species, the two remaining species are in the subgenus Trichanthidium, including a one-time discovered *Pachyanthidiumhimalayense* (Gupta & Sharma, 1993) and *Pachyanthidiumlachrymosum* (Smith, 1879) discovered from India and Chaiyaphum, Thailand (Bingham 1897; ITIS 2008; Tadauchi and Tasen 2009; Kumar et al. 2017).

#### ﻿ Pachyanthidium (Trichanthidium) lachrymosum

Taxon classificationAnimaliaHymenopteraMegachilidae

(Smith, 1879)

C8954D92-1B12-5387-B0E2-61BE6A8FD766

Fig. 12

Anthidium lachrymosum Smith, 1879: 463 (♀, ♂, syntype). from Bombay [Mumbai, Maharashtra, India] (NHMUK reg. number NHMUK014026059, examined).
Anthidium lachrymosum Smith: Bingham, 1897: 492.
Anthidium serapiforme Friese, 1914: 322 (♂). Holotype from Perak [Perak, Malaysia] (ZMB, not examined).
Pachyanthidium lachrymosum (Smith): ITIS 2008: http://www.itis.gov.
Pachyanthidium lachrymosum (Smith): Kumar et al. 2017: 452, 457–459, figs 15, 16.

##### Material examined.

(24♀, 3♂). **India**: 1♀, Bombay Dist. [= Mumbai, Maharashtra], B.M. TYPE HYM.17a 1866, (syntype) (NHMUK 014026059); Karnataka: Mysore, 1♀, 17 Apr. 2009, Dhanyavathi; “Yerbahalli” [must be Yerehalli, Mysore], 1♀, 29 Jul. 2014, Revanasidda (UAS); Bangalore, GKVK, 1♀, 29 Jul. 2014, Veereshkumar, 1♀, 25 Jun. 2014, Sunil, 1♀,18 Nov. 2014, Zameer, 2♂, 29 Apr. 2010, Arathi (UAS); Tamil Nadu: 1♀, Coimbatore, 5 Sep. 1950, P S Nathan (UAS); **Thailand**: 1♂, Chiang Mai (new record), Chom Thong District, Ban Luang Subdistrict, Doi Inthanon National Park, Ban Mae Klang Luang (18°32'29.7"N, 98°32'01.2"E, alt. 1,033 m), 7 May 2021, on *Bidenspilosa* (L.) [Asteraceae], T. Srimaneeyanon et al. (CUNHM: BSRU-AB-3551); 2♀, Kamphaeng Phet: Pang Sila Thong District, 7 Aug. 2015, N. Warrit et al. (CUNHM: BSRU-AA-4478, 4479); 6♀, Khon Kaen, Phu Wiang District, 26 May 2016, N. Warrit et al. (CUNHM: BSRU-AA-4484–4489); 1♀, Phayao, Mueang District, Phayao University (19°1'41.9334"N, 99°52'59.9730"E, alt. 493.41 m), 8 Oct. 2019, N. Warrit et al. (CUNHM); 8♀, Phetchabun, Namnao District, 19 Jun. 2017, N. Warrit et al. (CUNHM: BSRU-AA-4649–4656).

##### Distribution.

India (Karnataka (Bangalore, Mysore), Malabar (as per Bingham (1897), must refer to “Malabar coast” on southwest India), Maharashtra (Mumbai), Tamil Nadu (Coimbatore), Malaysia (Perak), Myanmar (Tenasserim), Thailand (Chaiyaphum, Chiang Mai (new record), Kamphaeng Phet (new record), Khon Kaen (new record), Phayao (new record), Phetchabun (new record)).

The records from Smith (1879) based on both sexes were noted to come from “Bombay district”, whereas Bingham (1897) who later revise Smith’s work, additionally mentioned “Malabar” and “Tenasserim” without any further information, also without any note if the additional material was examined. Most of the records from India are already listed by Kumar et al. (2017). Friese (1914) reported a male from Malaysia. In Thailand, Tadauchi and Tasen (2009) reported the species from Phu Khiao Wildlife Sanctuary in Chaiyaphum province.

##### Diagnosis.

*Pachyanthidiumlachrymosum* can be distinguished from other congeneric species by its black body with a white lateral band of short white hairs on the metasoma; lamellate parts are often translucent reddish brown to black; eyes with sparse short hairs; mandibles with four teeth; arolia absent; male similar to females but mostly differs in the presence of the arolia, three mandibular teeth, lateral spines on T3–T6, and a tridentate T7 (Fig. 12F, G), also noted in Bingham (1897) and Kumar et al. (2017). *Pachyanthidiumlachrymosum* also exhibits a robust, lamellate preoccipital ridge and an omaular carina, which are typical for *Pachyanthidium*.

##### Floral associations.

*Bidenspilosa* (L.) (Asteraceae) (this study), *Leucasaspera* (Willd.) Link (Lamiaceae) (Kumar et al. 2017).

##### Remarks.

The other four species of Pachyanthidium (Trichanthidium) were revised by Eardley and Griswold (2017). *Pa.lachrymosum* is the only species that does not have any integument maculation and has not been reported from the Afrotropical region. When compared with Indian specimens from both Kumar et al. (2017) and Smith (1879), we notice that the white hair patch on the metasoma appears to be more clumped and dense in Indian specimens, while in the Thai specimens the hairs seem looser and the patch more extended. The patch on the scutum is absent in some Thai individuals and white body patches are absent in some Indian specimens.

All specimens in CUNHM are females. Promisingly, two individuals are full of pollen trapped by the facial pubescence (Fig. 12A); thus, with their broad facial area, *Pa.lachrymosum* may also can gather pollen by rubbing it with its face (see also Portman et al. 2019; Kasparek et al. 2022).

Kumar et al. (2017) provides a note on Indian *Pa.lachrymosum* flight period (April, June, July, and November) and floral visitation (see above). Our specimens from Thailand were collected in May, June, and August.

#### ﻿ Pseudoanthidium

Taxon classificationAnimaliaHymenopteraMegachilidae

Friese, 1898

7A34ADAA-B1BF-5286-A2AA-5E9CF89EDB42

Anthidium (Pseudoanthidium) Friese, 1898: 101. Type species: Anthidiumalpinum Morawitz, 1873, designated by Sandhouse, 1943: 593. See Kasparek and Ebmer 2023.
Paranthidiellum Michener, 1948: 25. Type species: Anthidiumcribratum Morawitz, 1875, by original designation.
Pseudoanthidium (Paraanthidiellum) Pasteels, 1969: 79, unnecessary emendation of Paranthidiellum Michener.
Pseudoanthidium (Carinellum) Pasteels, 1969a: 80. Type species: Anthidiumochrognathum Alfken, 1932, by original designation.
Trachusa (Orientotrachusa) Gupta, 1993: 50. Type species: Anthidiumorientale Bingham, 1897, by original designation.
Pseudoanthidium Friese: Pasteels 1969: 76–77.

##### Note.

**Figure 13.**
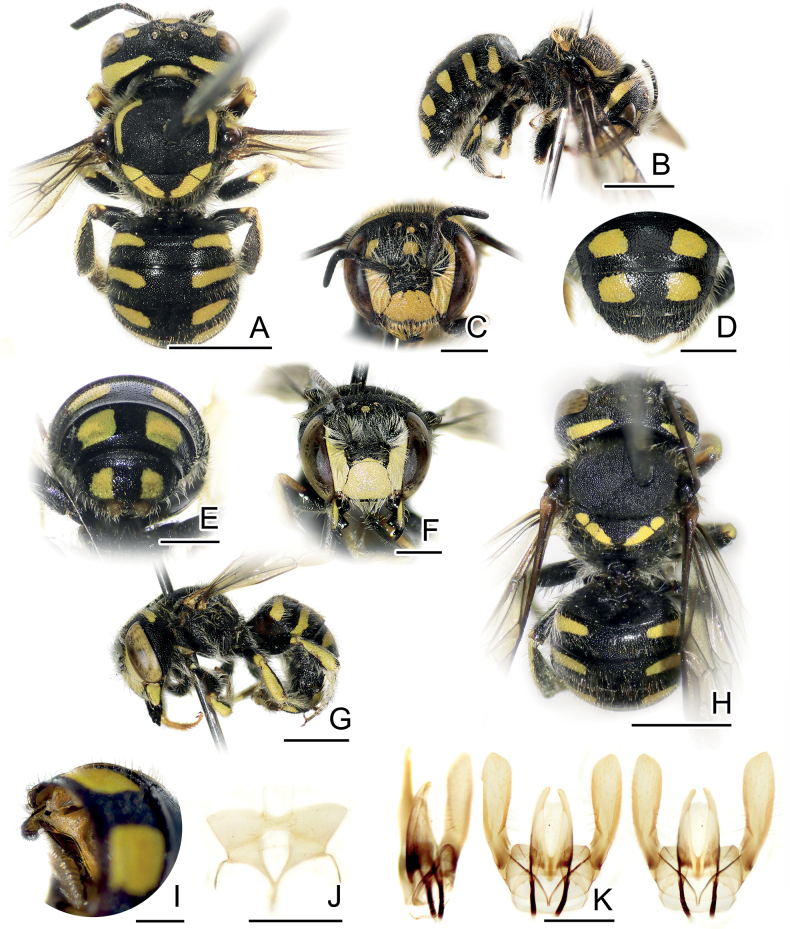
*Pseudoanthidiumorientale* (Bingham, 1897) [showing Mae Hong Son female (BSRU-AA-1240) (**A–D**), and male (BSRU-AA-1241) (**E–K**)] **A, H** dorsal habitus **B, G** lateral habitus **C, F** face **D** female T6 **E** male T7 **I** male S5 **J** S8 **K** male genitalia in lateral (left), dorsal (middle) and ventral (right). Scale bars: 2 mm (**A, B, G, H**); 1 mm (**C–F**); 0.5 mm (**I–K**).

*Pseudoanthidium* commonly has a tentorial pit placed below the connection of the subantennal suture and the epistomal suture (Michener 2007). The female mandible has more than four teeth, and the terga are without an apically depressed area (see Fig. 13E; Litman et al. 2016). As a very broad, ill-defined complex group, the South East Asian fauna is represented by only one subgenus, *Pseudoanthidium* s. str.

#### ﻿ Pseudoanthidium (Pseudoanthidium) orientale

Taxon classificationAnimaliaHymenopteraMegachilidae

(Bingham, 1897)

E622E175-5FAF-5C04-B223-9303ED116258

Figs 13 , 14A , 15

Anthidium orientale Bingham, 1897: 496 (♀). Holotype from Tenasserim, Myanmar, image examined in NHMUK under https://data.nhm.ac.uk/media/b2906d76-cd81-4776-9133-9558ce51baca.
Anthidium kryzhanovskii Wu, 1962: 167 (♀). Holotype from Jinping Xian, Yunnan, China (IZCAS: Institute of Zoology, Chinese Academy of Sciences, not examined).
Pseudoanthidium (Paraanthidiellum) orientale (Bingham): Pasteels 1969: 79, 80.
Trachusa (Orientotrachusa) orientale (Bingham, 1897): Gupta 1993: 55, 56, 58, figs 141–159 (♂ nov.).
Anthidium (s. str.) kryzhanovskii Wu, 1962: Wu 2006: 157 (♀, ♂).
Pseudoanthidium (Pseudoanthidium) orientale (Bingham): ITIS 2008: http://www.itis.gov.
Pseudoanthidium (Pseudoanthidium) orientale (Bingham): Niu et al. 2021: 139–142.

##### Material examined.

(10♀, 3♂). **Laos** (new record): 3♀, Champasak, Si Phan Don, Don Det, 20 Jan. 2015, N. Warrit et al., (CUNHM: BSRU-AA-1223, 1227, 1231); 1♀, Pakse, Bolaven plateau, Phu Suam Water Fall (15°16'44"N, 105°53'23"E), 19 Jan. 2015, N. Warrit et al., (CUNHM: BSRU-AA-1235); **Thailand**: Chiang Mai, 1♀, Mae Chaem District, Baan Na Jon (18°42'11.3970"N, 98°16'59.3754"E, alt. 874.93 m), 9 Dec. 2015, N. Warrit et al. (CUNHM: BSRU-AA-1243); 1♀, Chiang Mai, Chom Thong District, Doi Inthanon National Park, (18°32'12.39"N, 98°31'14.44"E, alt. 1,267 m), 16 Feb. 2021, Srimaneeyanon et al. (CUNHM: BSRU-AB-2970); 1♀, Kamphaeng Phet, Mueang District, (16°28'18"N, 99°29'43"E, alt. 10 m), 4 Dec. 2015, C. Thanoosing (CUNHM: BSRU-AA-1252); 1♂, Lampang, Mueang Pan District, Chae Son National Park (18°50'15.0498"N, 99°28'19.5594"E, alt. 451 m), TIGER project T-2922, Malaise trap, 8/14 Dec 2007, Boonruen & Acharaporn (CUNHM: BSRU-AB-4361); 2♀, 2♂, Mae Hong Son, Pang Tong, Under Royal Forest Park 2/ Pang Ung (19°29'58.3008"N, 97°54'42.1014"E, alt. 1,164 m), 10 Dec. 2015, N. Warrit et al. (CUNHM: ♀ BSRU-AA-1239, 1240, ♂ BSRU-AA-1241, 1242); 1♀, Phayao, Mueang District, Maeka Subdistrict, Phayao University (CUNHM: BSRU-AA-1238, Phayao University).

##### Records from iNaturalist

**(2023).** Thailand: Chiang Mai, San Sai District, San Sai Noi Subdistrict (18°49'08.6"N, 99°01'15.1"E) uploaded by ‘jackychiangmai’ on 14 Jan. 2022 (observation id:104911660); Chiang Rai, Chiang Saen Lake, Viang Yonok Hotel (20°15'42.5"N, 100°02'59.5"E), uploaded by ‘pam-pilombino’ on 27 Jan 2020.

##### Distribution.

Cambodia (Mondulkiri: Ascher et al. 2016), China (Yunnan), India (Alwar, Poona, Solan, Tolawas, Udaipur), Laos (Champasak (new record), Myanmar (Tenasserim), Thailand (Chiang Mai, Chiang Rai (new record from iNaturalist 2023), Kamphaeng Phet (new record), Lampang (new record), Lamphun (new record from iNaturalist 2023), Mae Hong Son (new record), Phayao (new record)). For the Indian records from Gupta (1993), see Remarks section below.

##### Diagnosis.

*Pseudoanthidiumorientale* is a medium-sized bee (6–8 mm) and usually has a black integument with yellow maculations in all tagmata. It has a remarkably pale yellow mark on the paraocular area reaching close to the top of eyes, female mandibles with five or six teeth, rounded scutellum with broad marginal maculation which is medially disrupted, tibia and tarsi yellow except black on the venter, female terga with yellow paramedian maculation on T1–T5 in female, which is more laterally extended on T1 and T2 and nearly rectangular in T3–T5. The male looks superficially similar to the female but has a different dentition of the mandible (i.e., distinctly tridentate, broader especially the inner tooth), and maculation on T6 and T7. Male genitalia broad.

##### Floral associations.

Plants with hairy surfaces (see iNaturalist observation from Lamphun) must be the resources for the nesting material. Also, from iNaturalist image from Chiang Mai, the photographs clearly show the bee wandering on the inflorescence of *Antigononleptopus* Hook. & Arn. (Polygonaceae), which is a hairy plant, although there is no direct evidence to this claim. Niu et al. (2021) also noted the floral associations for *Ps.orientale* including *Blumea* sp. (Asteraceae), *Eupatoreae* sp. (Asteraceae), *Helianthusannuus* L. (Asteraceae), *Tephrosiapurpurea* (L.) Pers. (Fabaceae; originally noted as *Tephrosiahamiltoni*), and *Tridax* sp. (Asteraceae; originally noted as “*Tridex*”).

##### Remarks.

This species was described by Bingham (1897) as *Anthidiumorientale*, based on a female from Tenasserim, Myanmar. Pasteels (1969) then assigned it to Pseudoanthidium (Paraanthidiellum) Michener, 1948. Gupta (1993) provided both female and male descriptions and illustrations from India, which is designated as Trachusa (Orientotrachusa) orientale. Later, the species was listed as Ps. (Pseudoanthidium) in the World Bees Checklist Project (ITIS 2008). A female *Ps.orientale* was reported from a monochrome image from Chiang Mai, Thailand (Tadauchi and Tasen 2009), and a male was reported from Bousra, Mondulkiri, Cambodia (Ascher et al. 2016) with photographs. However, the CUNHM male specimens studied here are apparently different from the male descriptions and photographs from Cambodia in lacking the antero-lateral maculation on the scutum. Gupta’s (1993) male description from India also stated that “tergum first with a complete band, T2 and T3 interrupted medially, T4–T7 entirely yellow” which is incongruent to the CUNHM specimens studied here, and also to the photographs of the male from Cambodia; therefore, the Indian specimens need further study.

**Figure 14.**
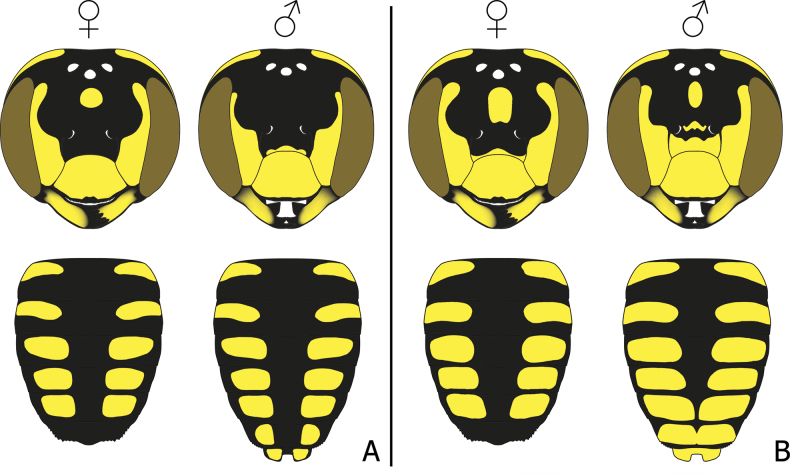
Facial and metasomal illustrations of **A***Pseudoanthidiumorientale* (Bingham, 1897) and **B***Ps.rotundiventre* (Pasteels, 1980).

*Pseudoanthidiumorientale* is superficially similar to *Ps.rotundiventre* (Pasteels, 1987) from Sri Lanka and India. Kumar et al. (2017) mentioned that these two species were probably referred to the same species. As most of their distinct morphology is similar, the difference is based on color (Fig. 14). First, *Ps.rotundiventre* has a more extended maculation on the face, the female has an entirely yellow clypeus, and a W-shape maculation on the supraclypeal area. Seven Thai females of *Ps.orientale* come without supraclypeal mark and show reduction of the clypeal color as a black stigma, while the remaining two from Laos have an entirely yellow clypeus and the supraclypeal mark is only a triangle in the median part. Second, it is remarkable that *Ps.rotundiventre* comes with more extended maculation on the male terga: the median interruption of the band tends to decrease from base to apex on the metasoma. Specifically, T5 and T6 are nearly uninterrupted and T7 is entirely yellow. The *Ps.orientale* males show much more consistency in the median interruption of the tergal maculation, making it appear similar to the female. Hence, Gupta’s *Ps.orientale* male description from India is more congruent to *Ps.rotundiventre*.

As the male genitalia of *Ps.orientale* illustrated in Gupta (1993: fig. 159) is inadequate to compare with microphotographs of the male genitalia of *Ps.rotundiventre* (see Kumar et al. 2017: fig. 20C), we used a microphotograph of Thai *Ps.orientale* genitalia which was prepared and preserved in glycerin. Overall, the genitalia seem to be identical, but the photograph of the genitalia of *Ps.rotundiventre* is not clear, so the shape and sclerotization of the apodeme of penis valve is hard to compare with that of *Ps.orientale* (cleared one in Fig. 13K). However, the respective characters do not seem to have much of value in sexual differentiation.From the evidence, it seems that color variation in *Pseudoanthidiumorientale* is variable, and potentially similar to *Ps.rotundiventre*. At the same time, *Ps.flaviventre* Cameron, 1897, belonging to the same subgenus, displays much more obvious differences in male genitalia and other external body parts (also see Kumar et al. 2017). Because of the similarities, Kumar et al. (2017) suggested that *Ps.rotundiventre* is a junior synonym of *Ps.orientale*, but the types need to be examined before making a final decision.

Finally, there is an observation of wool-collecting behavior of a female of *Ps.orientale* from Chiang Saen Lake, Chiang Rai, Thailand, reported and observed by Ms. Pamela Piombino [user: ‘pam-pilombino’] on 27 January 2020, and published on iNaturalis.org (iNaturalist 2023). Based on personal communication, the bees landed on a cushion with a mouthful of fiber-ball, wandered around for a few minutes, and eventually flew off without any “active” fiber-collecting behavior. We are not sure if this fiber was collected precisely from the cushion or elsewhere, but this is the first time that fiber-collecting behavior has been observed in this species.

**Figure 15.**
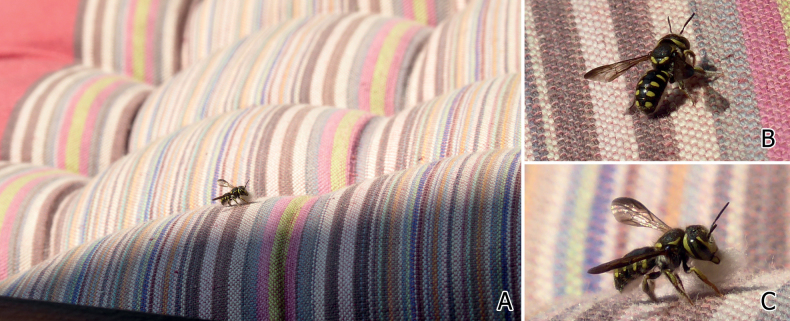
*Pseudoanthidiumorientale* wandering around on a cushion with a fiber ball (**A–C** for cropped close-up). Original photographs taken by Pamela Piombino.

#### ﻿ Stelis

Taxon classificationAnimaliaHymenopteraMegachilidae

Panzer, 1806

4BFFD145-2C26-5DD1-98ED-6D9238EBFA2F

Trachusa Jurine, 1801: 164 (nec Panzer 1804). Type species: Apisaterrima Panzer, 1798, by designation of Morice and Durrant 1915: 426. Suppressed by Commission Opinion 135, 1939 (Direction 4).
Stelis Panzer, 1806: 246. Type species: Apisaterrima Panzer, 1798 (nec Christ 1791) = Apispunctulatissima Kirby, 1802, monobasic.
Gyrodroma Klug in Illiger 1807: 198; Klug 1807: 225. Type species: Apisaterrima Panzer, 1798 (not Christ 1791) = Apispunctulatissima Kirby, 1802, designated by Sandhouse 1943: 555. [Sandhouse incorrectly considered Gyrodroma to be monobasic; two species were listed by Klug in Illiger 1807, which has page priority over Klug 1807].
Gymnus Spinola, 1808: 9. Type species: Apisaterrima Panzer, 1798 (nec Christ 1791) = Apispunctulatissima Kirby, 1802, monobasic.
Ceraplastes Gistel, 1848: x [10], unjustified replacement for Stelis Panzer, 1806. Type species: Apisaterrima Panzer, 1798 (nec Christ 1791) = Apispunctulatissima Kirby, 1802, autobasic.
Chelynia Provancher, 1888: 322. Type species: Chelynialabiata Provancher, 1888, monobasic [see Provancher 1889].
Melanostelis Ashmead, 1898: 283. Type species: Melanostelisbetheli Ashmead, 1898 = Stelisrubi Cockerell, 1898, by original designation.
Stelidium Robertson, 1902: 323. Type species: Stelidiumtrypetinum Robertson, 1902, monobasic [see Michener 1997].
Microstelis Robertson, 1903: 170, 175. Type species: Stelislateralis Cresson, 1864, by original designation.
Stelis (Pavostelis) Sladen, 1916: 313. Type species: Stelismontana Cresson, 1864, monobasic.
Stelis (Stelidina) Timberlake, 1941: 131. Type species: Stelishemirhoda Linsley, 1939, by original designation.
Stelis (Stelidiella) Timberlake, 1941: 133. Lapsus for Stelidina Timberlake, 1941.
Stelis (Leucostelis) Noskiewicz, 1961: 126, 132. Type species: Gyrodromaornatula Klug, 1807, by original designation.

##### Note.

Most of the cleptoparasitic bees of the Anthidiini are attributed to the genus *Stelis* due to the very diverse morphs. The recent works by Michener and Griswold (1994), Michener (2000, 2007), and Kasparek (2015) for species in Europe, North Africa, and the Middle East provide comprehensive information for *Stelis*. Female *Stelis* notably lack scopa and juxta-antennal carina, while the carinae on prosoma and mesosoma can be absent or weakly present. In males, T7 is round, weakly bilobed, or trilobed. The only subgenus discovered in Thailand is *Malanthidium* (see Nalinrachatakan 2021b), only known by males and can be recognized by its distinct postero-lateral hook on its axilla.

#### ﻿ Stelis (Malanthidium) flavofuscinular

Taxon classificationAnimaliaHymenopteraMegachilidae

Nalinrachatakan & Warrit, 2021

10B0D392-12FC-5B4B-BC70-A3B5D1F017EE

Fig. 4C

Stelis (Malanthidium) flavofuscinular Nalinrachatakan & Warrit in Nalinrachatakan et al. 2021b: 172–175, see figs 6, 7. (♂) Holotype and paratype from Phu Chong Na Yoy National Park, Ubon Ratchathani, Thailand (CUNHM).

##### Material examined.

(2♂). Same specimens as in Nalinrachatakan et al. (2021b). Holotype transferred to NHMUK in April 2023.

##### Distribution.

Thailand (Ubon Ratchathani: Phu Chong Na Yoy National Park).

Since *Stelis* is a cleptoparasitic bee, its distribution must be in accordance with its host. Noteworthy, the other known species of the subgenus Malanthidium, *S.macaccensis* (Friese, 1914) is known from Malaysia; thus, Malanthidium is the only subgenus of Stelis present in South East Asian region.

##### Bee host.

*Anthidiellumphuchongense* Nalinrachatakan & Warrit, 2021.

##### Floral association.

Unknown.

##### Remarks.

With only two males known, some differences between both specimens and their biology were mentioned and discussed in Nalinrachatakan et al. (2021b).

#### ﻿ Trachusa

Taxon classificationAnimaliaHymenopteraMegachilidae

Panzer, 1864

B2CF1F65-B8D2-5B08-AB0C-EFDE4D35354D

Trachusa Panzer, 1804: 14–15. Type species: Trachusaserratulae Panzer, 1804 = Apisbyssina Panzer, 1798, by designation of Sandhouse 1943: 605.
Diphysis Lepeletier, 1841: 307. Type species: Diphysispyrenaica Lepeletier, 1841 = Apisbyssina Panzer, 1798, monobasic.
Megachileoides Radoszkowski, 1874: 132. Type species: Trachusaserratulae Panzer, 1804 = Apisbyssina Panzer, 1798, by designation of Michener 1995: 375.
Megachiloides Saussure, 1890: 35, incorrect spelling of Megachileoides Radoszkowski, 1874; see Michener 1995.

##### Note.

A medium to large, robust, round-edged species, genus *Trachusa* appears to be sister to the remainder of the tribe Anthidiini (Litman et al. 2016). Recently, Kasparek (2017, 2019a) reviewed the old-world *Trachusa* and described new Malaysian species, respectively.

#### ﻿ Trachusa aff. vietnamensis

Taxon classificationAnimaliaHymenopteraMegachilidae

Flaminio & Quaranta, 2021

18C2F781-0391-55F4-B69C-7F8A18BCE675

Figs 16 , 17

Trachusa vietnamensis Flaminio & Quaranta in Flaminio et al. 2021: 307–310, fig. 1 (♀). Holotype from Quang Nam, Vietnam (CREA: Consiglio per la ricerca in agricoltura e l’analisi dell’economia agrarian (Bologna, Italy), examined).

##### Material examined.

(5♀). **Thailand**: Phitsanulok, Nakhon Thai District, 27 May 2014, N. Warrit et al. (CUNHM: BSRU-AA-4471–4475).

##### Distribution.

Thailand (Phitsanulok) and Vietnam (Quang Nam).

##### Diagnosis.

The species is very close to, or maybe identical to *Trachusavietnamensis*. Only the female is known: body large, robust, and black. Bands with yellowish, orangish, or light-brown coloration on the vertex, preoccipital area, anterolateral of scutum, and scutellum, while mesosoma covered in orangish pubescence. The Thai specimens are distinguished from the Vietnamese material by a unique elongate metasoma making it more chalicodomiform, and more limited maculation on the metasoma (fully striped on T1 and T2 of *T.vietnamensis*, small pale marks on the side of T1 and T2 and minute or absent on T3 and T4 for Thai specimens). The species is also close to *T.ovata*, from which it differs by the combination of five mandibular teeth, clypeus black with ill-defined shiny median longitudinal line, conspicuous rounded light-brown scutellum which seems darker basally, and head with orangish to light-brown maculations running continuously from the vertex to genal area.

##### Description.

**Female**: Body length 13.4, 13.3, 13.2, 13.0, 13.3 mm, head width 4.3, 4.3, 4.2, 4.3, 4.1 mm, intertegular distance 3.6, 3.8, 3.8, 3.7, 3.5 mm, respectively. Wingspan 25.0, 25.6, 25.0, 24.7, 24.9 mm.

**Figure 16.**
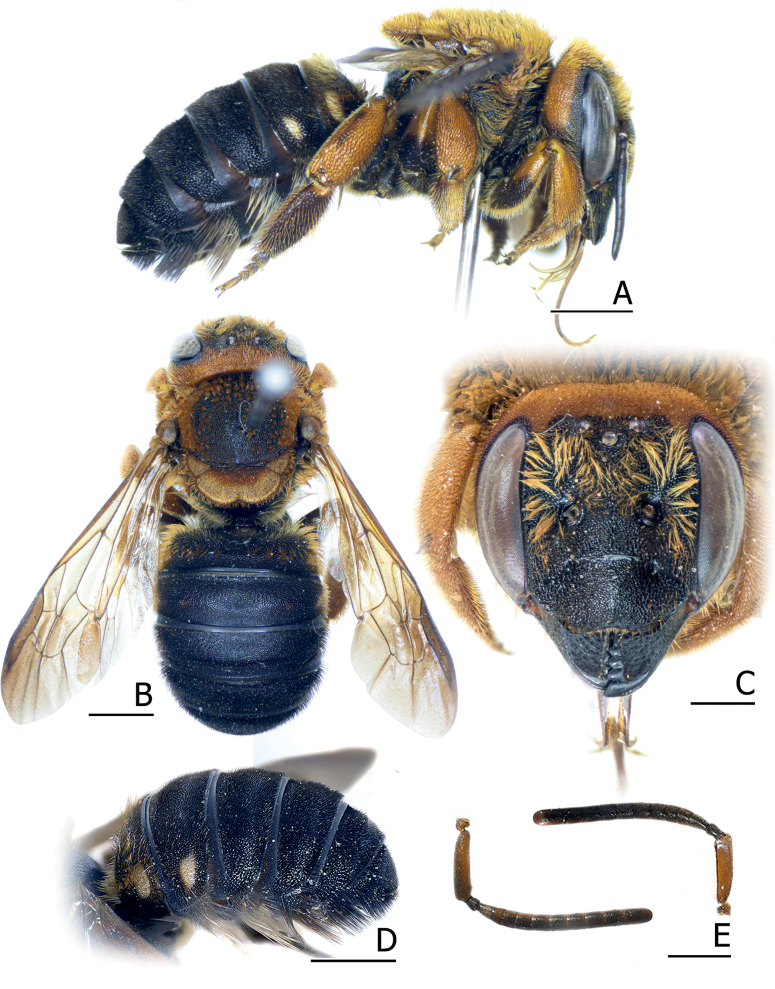
Female Trachusaaff.vietnamensis BSRU-AA-4473 (**A**), 4744 (**B–E**) **A** lateral habitus **B** dorsal habitus **C** face **D** lateral of metasoma **E** antennae. Scale bars: 2 mm (**A, B, D**); 1 mm (**C, E**).

Head largely black, with light-brown band on vertex running continuously to genal area, lighter on occipital ridge, but not abutting margins of eyes and ocelli. Clypeus (see Fig. 16C) black, slightly convex but shallow depression apically with ill-defined shiny median longitudinal line; middle and apical area with smaller and denser punctures, apical margin slightly crenulate thus looking somewhat emarginate at middle. Surfaces apically covered with sparse short yellow hair that is dense and long. Supraclypeal area black, slightly convex, with shiny median longitudinal line. Mandible broad with apex ~ 1.5× wider than base. Mandibles somewhat dull, black with light-brownish mark on basal area, inner surfaces with hairs. Four mandibular teeth, apical tooth larger than inner. Labrum black, with yellow bristles on its apex. Maxillary palpus three-segmented, black but dark brown on rounded basal segment. Subantennal suture slightly arcuate outwards. Antenna generally brown, scape brown but darker on front, pedicel dark brown to black, flagellum lighter in color on medial front, F1 and F2 brighter than rest. Ocellooccipital distance not more than 1.5× interocellar distance. Head covered with fulvous hair, denser and longer on frons, paraocular area, and lower part of genal area.

Mesosoma black, covered with fulvous hair except on pronotum with exposed shiny black median area with coarse punctures. Pronotal lobe strongly carinated, light brown. Mesepisternum black. Omaulus carinated, extending to ventral part of thorax. Scutum laterally carinate, punctures uniform, dense, with light-brown color on anterolateral band, not abutting together in middle. Axilla rounded laterally, entirely light brown. Tegula fulvous with dark patch lining medio-posterior. Scutellum broad, apically round with median emargination, light brown, darker on median triangular basal area.

Wing subhyaline, fuscate, forewing darker at apical margin and marginal cell. Pterostigma brown. Veins dark brown to black; 2^nd^ recurrent vein abutted to 2^nd^ submarginal crossvein distally.

Legs covered with short fulvous hairs. Coxae, trochanters, and basal parts of femora dark reddish brown to black; legs otherwise light brown except dark brown on inner surfaces of basitarsus and tarsi, slightly subtle on outer surfaces of hind basitarsus and hind tarsal segments. Apical tarsal segments with apical dark spot. Claw with inner tooth, light brown, apically black. Arolium present, dark brown to black.

**Figure 17.**
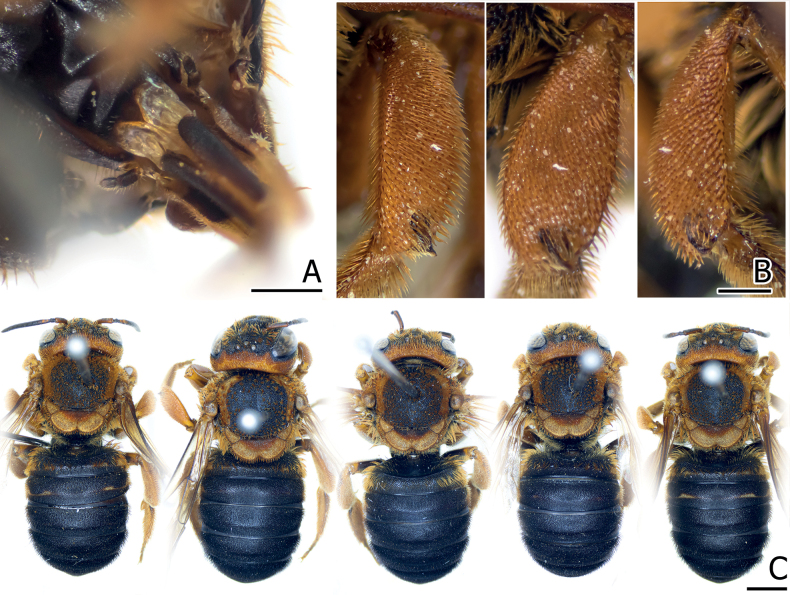
Female Trachusaaff.vietnamensis (BSRU-AA-4475) **A** ventral habitus of mouthparts (with removed mandible) **B** mid tibia **C** all specimens in dorsal view, showing variation (BSRU-AA-4471–4475, respectively from left to right). Scale bar: 2 mm (**C**); 0.5 mm (**A, B**).

Metasoma black. Discs of all terga swollen, with fine dense punctures. Terga covered with short black hairs, lighter to fulvous hairs on T1–T3 lateral surfaces, longer fulvous hairs covering frontal carina of T1. T1 and T2 with small pale lateral patches (Figs 16A, B, D, 17C) with part of obscured thin line extending to median. In some paratypes, these patches can be more or less expressed, tending to form an almost continuous thin but obscure band on disc, and also obscurely found on T3 and T4 in one specimen (BSRU-AA-4471). Scopa pale yellow on S2 and S3, gradually darker on S4, and becoming black on S5 and S6. S1–S6 reddish brown to black, darker apically. T6 with barely visible small median emargination.

##### Remarks.

*Trachusa* species have been reported from upper and lower Indochina but with limited materials (Soh et al. 2016; Kasparek 2017, 2019a; Flaminio et al. 2021). It is plausible that the lack of previous records in Thailand, Burma, and Cambodia may be due to limited collecting, in addition to the general rareness of *Trachusa* bees. This is the first record of *Trachusa* in Thailand: all specimens are neither complete nor in perfect condition.

Since the species is very close to *T.vietnamensis* from Vietnam, here we propose that the Thai specimens belong to the same species. The differences in tergal bands on the metasoma may be considered as variation; however, the Thai specimens exhibit a more elongate metasoma. To confirm that both species are indeed the same, DNA barcoding would be useful since the barcode of *T.vietnamensis* was provided by Flaminio et al. (2021).

Subgeneric placement of *Trachusavietnamensis* is still uncertain. Kasparek (2017, 2019a) assigned three species groups in *Paraanthidium* with their characteristics: *interrupta* group (female with bright yellow maculations on black, wasp-like), *longicornis* group (Indomalayan species with female having dull yellow maculation except on the mesosoma), *ovata* group (female completely without maculation), and the remaining *Trachusaxylocopiformis* (Mavromoustakis, 1954), for which only the male is known Fukien, China, is large and black except for yellow on lower part of the face.

*Trachusavietnamensis* seems to not be congruent with any of these groups, but is closely related to the *ovata* group by its face, especially in its clypeal shape, and the reduced maculation on the metasoma. Also, the superficially color pattern and almost parallel-sided body form are not congruent with the robust-megachiliform that occurs in all described females of Paraanthidium ; from this, it more resembles the subgenus Orthanthidium from mainland China and Taiwan for which two fairly different species are known: *Trachusaformosana* (Friese, 1917) and *T.cornopes* Wu, 2004. *Orthanthidium* was designated by Mavromoustakis (1953) prominently for its parallel-sided axilla, truncated scutellum, and small spine on the tibial apex. As *Orthanthidium* is still problematic in its status (Kasparek 2017), future work is still needed.

The astonishing record of another *Trachusa* species that is completely different from the aforementioned T.aff.vietnamensis has been retrieved from the citizen science database platform iNaturalist (iNaturalist 2023) from Thailand: Chiang Mai, Mueang District, Suthep Subdistrict, Doi Suthep-Pui (18°49'00.5"N, 98°55'26.8"E), observed repeatedly by “jackychiangmai” on 16 Apr, 30 Apr, and 27 May 2022 (observation id: 111730798, 114167310, 119204705 respectively).

**Figure 18.**
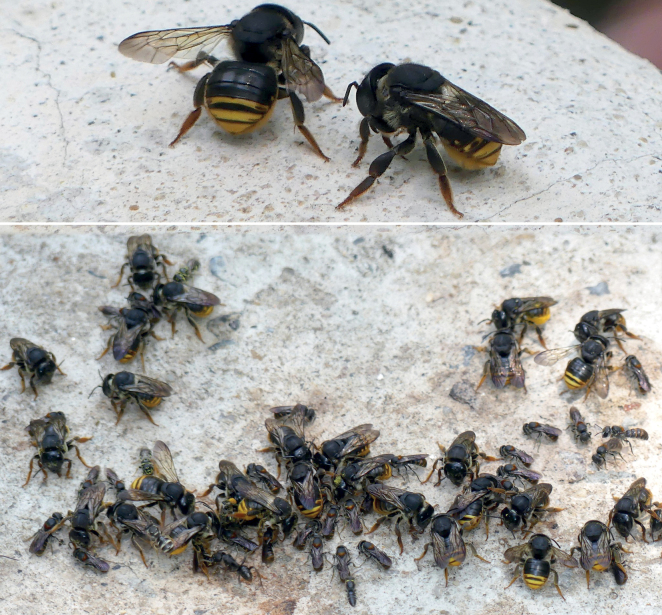
Groups of Trachusa (Paraanthidium) sp. primarily identified as *longicornis* group sensu Kasparek (2017), gather with other bee species. Photograph: Jacky Cudon.

Since the identification is restricted to the available photographs, we cannot identify the bee definitively. These observations show multiple *Trachusa* bees (25+) grouping on a semi-limestone concrete surface (Fig. 18) along with other bees, including Ceratina (Ceratinidia) spp., *Chelostomaaureocinctum* (Bingham, 1897), possibly *Hylaeus* sp., and a Halictinae bee (possibly *Pachyhalictus* or *Lasioglossum*). This *Trachusa* species exhibits a large robust body with a round scutellum, while the yellow band on T4–T6 and the yellowish brown patch apically on the leg can also be noticed. Most bees that can be speculated for their sex are usually female based on their pollen-loaded metasomal scopa. Based on the available information, we classify these Trachusa bees to the subgenus Paraanthidium, primarily within *longicornis* group that contains four described species at present: *T.longicornis* (Friese, 1902), *T.maai* (Mavromoustakis, 1953), *T.muiri* (Mavromoustakis, 1937), and *T.rufobalteata* (Cameron, 1902) (see discussion above and Kasparek 2017). Collections and information are needed in order to confirm its identity.

**Keys to the species of Anthidiine bees in Thailand**

Two keys are provided below, one for females and one for males. The keys are modified from Baker (1995), Michener (2007), Engel (2009), Niu et al. (2019), and Flaminio et al. (2021). They both exclude morphospecies known only from citizen science records.

### ﻿Key to females of anthidiine bees in Thailand

Excluding *Stelisflavofuscinular* as the female is unknown but must presumably be identifiable to genus due to the absence of metasomal scopa and juxta-antennal carina.

**Table:** 

1	Mandible with ≥ 4 teeth. Terga without depressed apical zone (genus *Pseudoanthidium*); body black with distinct yellow maculation, especially lateral yellow patch on all terga except T6	***Pseudoanthidiumorientale***
–	Mandible teeth < 4 teeth. Terga with apical zone either depressed or not depressed. Terga without yellow maculation but, if present, the pattern will differ from above	**2**
2	Face with both longitudinal median carina and juxta-antennal carina (Figs 9B, 10B, F). Metasomal scopa absent (genus *Euaspis*)	**3**
–	Face without carinae as described above. Metasomal scopa present	**6**
3	S6 acute with median carina and lateral tooth (Fig. 10D). Scutellum extended with medial shallow depression, black without pale maculation on the margin	***Euaspispolynesia***
–	S6 broad, obtuse, or subacute, with a basal platform. Scutellum black with pale maculations on the margin	**4**
4	S6 with distinct basal platform (Fig. 9C, E). Clypeal punctures irregular, with a strong distinct median carina (Fig. 9B)	***Euaspisaequicarinata***
–	Basal platform of S6 not distinct but can be noticed at median area. Clypeus without median carina while punctation somewhat irregular	**5**
5	S6 apical margin obtuse, basal platform arise as a bulge on the median area (Fig. 10H)	***Euaspisstrandi***
–	S6 apical margin subacute, basal platform smaller (Fig. 11D, E). In *Euaspiswegneri* sensu Baker (1995: fig. 31), punctures on scutum and especially on scutellum finer and denser (larger in Thai specimens, see Fig. 10B)	**Euaspisaff.wegneri**
6	Face with a pair of juxta-antennal carinae but without longitudinal median carina (Fig. 6B, E–G). (genus *Eoanthidium*); subantennal suture straight	***Eoanthidiumriparium***
–	Face without any distinct carina. Subantennal suture arcuate	**7**
7	Large species (length > 11 mm). Cu-V of hindwing usually ≥ half of 2^nd^ M+Cu. (genus *Trachusa*)	**Trachusaaff.vietnamensis**
–	Smaller (< 11 mm long). Cu-V of hindwing < half of 2^nd^ M+Cu	**8**
8	Omaular carina not extending down to the venter of thorax (genus *Bathanthidium*); Paraocular area black. T6 with median raised platform (similar to Fig. 5C)	***Bathanthidiumbinghami***
–	Omaulus with a distinct carina, extended to the venter of thorax. T6 without raised platform	**9**
9	Preoccipital ridge and omaulus lamellate (Fig. 12E) (genus *Pachyanthidium*). Body black. Metasomal terga with basolateral white hair patches. Arolia absent	***Pachyanthidiumlachrymosum***
–	Omaulus carinated but not lamellated (genus *Anthidiellum*). Body black with yellow maculations, or reddish to fulvous. Metasoma without clumping white hair patches; arolia present	**10**
10	Body black with distinct yellow maculations scattered in most parts. The apex of mandible little wider than its base. T1 with obvious anterior carina which separates frontal and dorsal surfaces	**11**
–	Body somewhat orangish to fulvous, or black. If black, without distinct yellow maculations on metasoma. Apex of mandible ~ 1.5× wider than its base. T1 without distinct carina	**12**
11	Small species (length ~ 4–5 mm). Hind tibia and basitarsus simple without any distinct swollen parts. T1 with lateral yellow patches. T2 black while T3–T6 with yellow transverse band which is often medially disrupted on T3 (see Fig. 1)	***Anthidiellumsmithii***
–	Moderate species (length ~ 7 mm). Hind tibia and basitarsus distinctly enlarged. Yellow marks present on each tergum, medially disrupted on T1–T3, and becoming full stripes on T4–T6 (see Fig. 2)	**Anthidiellumaff.latipes**
12	Head extensively black, brownish on clypeus and lower part of paraocular area. Scutum black. Metasoma dark brown to black, with metallic red infused especially on T2 and T3	***Anthidiellumapicepilosum***
–	Head orange or fuscous, without extensive black maculation; if present, only on frons. Scutum reddish or fulvous, with extensive black marks. Metasoma reddish or fulvous, sometimes with black marks	**13**
13	Body largely ferruginous. T6 black, covered with golden-white hairs. T1–T5 sometimes with scattered black maculations (Fig. 4A)	***Anthidiellumignotum***
–	Body appears reddish to orange. T6 orange while T1–T5 have a black apical band (Fig. 4B)	***Anthidiellumphuchongense***

### ﻿Key to males of anthidiine bees in Thailand

Excluding the males of Anthidiellumaff.latipes, Euaspisaff.wegneri, and Trachusaaff.vietnamensis, as they are unknown. Also note that the status of male *Euaspisaequicarinata* and *Eu.strandi* is still ambiguous.

**Table:** 

1	Arolia absent. Preoccipital ridge and omaulus not carinate. Terga without depressed apical zone (genus *Pseudoanthidium*). Body black with yellow maculation. S3 with apical extended lobe, lined with a series of yellow hair fringes (Fig. 13I)	***Pseudoanthidiumorientale***
–	Arolia present. Preoccipital ridge smooth or carinated, omaulus carinated, or at least in the dorsal part. Body black with yellow maculation, or different. S3 without extended apical lobe	**2**
2	Face with both longitudinal median carina and juxta-antennal carinae (as Figs 9B, 10B, F) (genus *Euaspis*)	**3**
–	Face without combination of carinae as in above	**5**
3	Scutellum extended with distinct medial shallow depression, black. Apical lamina of gonoforceps with length < 2× its width	***Euaspispolynesia***
–	Scutellum apically with small median notch, black, with or without pale maculation on the margin. Apical lamina of gonoforceps length > 2× its width	**4**
4	Clypeal punctation irregular, with a strong median carina. Scutellum black with pale maculation on the margin	***Euaspisaequicarinata* sensu Pasteels (1980)**
–	Clypeus without median carina while the punctures only somewhat irregular. Scutellum black without pale maculation on the margin	***Euaspisstrandi* sensu Baker (1995)**
5	Face with a pair of juxta-antennal carinae but without longitudinal median carina (Fig. 6B, E–G). (genus *Eoanthidium*); Subantennal suture strait	***Eoanthidiumriparium***
–	Face without distinct carina. Subantennal suture arcuate	**6**
6	Front and middle tibia with two apical spines (genus *Stelis*). Body elongate. Axilla with yellow posterolateral hook	***Stelisflavofuscinular***
–	Front and middle tibia with one apical spine. Body robust, not elongate. Axilla without posterolateral hook	**7**
7	Omaular carina incomplete, not extending down to the venter of thorax (genus *Bathanthidium*); Paraocular area black. T6 and T7 with median raised platform (Fig. 5C)	***Bathanthidiumbinghami***
–	Omaulus distinctly with complete carina. Terga without median raised platform	**8**
8	Preoccipital ridge and omaulus carinate or lamellate (Fig. 12E) (genus *Pachyanthidium*); Body black. Metasomal terga with basolateral white hair patches	***Pachyanthidiumlachrymosum***
–	Omaulus carinate but not lamellate (genus *Anthidiellum*). Body black with yellow maculations, or reddish to fulvous. Metasoma without basolateral white hair patches	**9**
9	Small species (< 6 mm). Body black with yellow maculation. T1 with obvious anterior carina and lateral yellow maculation. T2 black while T3–T7 with yellow transverse band often medially disrupted on T3	***Anthidiellumsmithii***
–	Larger species (usually > 6 mm). Body somewhat orangish to fulvous. T1 without frontal carina	**10**
10	Scutum black. Metanotum black to dark ferruginous, brighter in T5–T7	***Anthidiellumapicepilosum***
–	Scutum and metanotum reddish or fulvous, with black marks infused	**11**
11	Body integument largely ferruginous. Face with extensive black marks. S4 gradulus incomplete. Apical lamina of gonoforceps without inner apical angulation	***Anthidiellumignotum***
–	Body integument appears orangish. Facial black mark restricted to the frons. S4 gradulus complete. Apical lamina of gonoforceps with inner apical angulation	***Anthidiellumphuchongense***

## Supplementary Material

XML Treatment for Anthidiellum

XML Treatment for Anthidiellum (Pycnanthidium) smithii

XML Treatment for Anthidiellum (Pycnanthidium) latipes

XML Treatment for Anthidiellum (Ranthidiellum) apicepilosum

XML Treatment for Anthidiellum (Ranthidiellum) ignotum

XML Treatment for Anthidiellum (Ranthidiellum) phuchongense

XML Treatment for Bathanthidium

XML Treatment for Bathanthidium (Manthidium) binghami

XML Treatment for Eoanthidium

XML Treatment for Eoanthidium (Hemidiellum) riparium

XML Treatment for Euaspis

XML Treatment for Euaspis aequicarinata

XML Treatment for Euaspis polynesia

XML Treatment for Euaspis strandi

XML Treatment for Euaspis aff. wegneri

XML Treatment for Pachyanthidium

XML Treatment for Pachyanthidium (Trichanthidium) lachrymosum

XML Treatment for Pseudoanthidium

XML Treatment for Pseudoanthidium (Pseudoanthidium) orientale

XML Treatment for Stelis

XML Treatment for Stelis (Malanthidium) flavofuscinular

XML Treatment for Trachusa

XML Treatment for Trachusa aff. vietnamensis

## ﻿Additional information

### Author ORCIDs

Pakorn Nalinrachatakan https://orcid.org/0000-0001-7962-5844

John S. Ascher https://orcid.org/0000-0002-7887-2461

Max Kasparek https://orcid.org/0000-0002-5604-6791

Prapun Traiyasut https://orcid.org/0000-0002-7114-0890

Chawatat Thanoosing https://orcid.org/0000-0002-4228-748X

Natapot Warrit https://orcid.org/0000-0002-6338-1782

### Data availability

All of the data that support the findings of this study are available in the main text.
